# PD‐1‐induced T cell exhaustion is controlled by a Drp1‐dependent mechanism

**DOI:** 10.1002/1878-0261.13103

**Published:** 2021-10-14

**Authors:** Luca Simula, Ylenia Antonucci, Giorgia Scarpelli, Valeria Cancila, Alessandra Colamatteo, Simona Manni, Biagio De Angelis, Concetta Quintarelli, Claudio Procaccini, Giuseppe Matarese, Claudio Tripodo, Silvia Campello

**Affiliations:** ^1^ Department of Biology University of Rome Tor Vergata Rome Italy; ^2^ Tumor Immunology Unit Department of Health Sciences University of Palermo School of Medicine Palermo Italy; ^3^ Department of Molecular Medicine and Medical Biotechnologies University of Naples Federico II Naples Italy; ^4^ Department of Onco‐Hematology and Oncology, Cell and Gene Therapy IRCCS Bambino Gesù Children’s Hospital Rome Italy; ^5^ Department of Translational Medical Sciences University of Naples Federico II Naples Italy; ^6^ Institute for Endocrinology and Experimental Oncology “G. Salvatore” CNR Naples Italy; ^7^ IRCCS Santa Lucia Foundation Rome Italy; ^8^ Histopathology Unit FIRC Institute of Molecular Oncology (IFOM) Milan Italy; ^9^ Present address: Department of Infection, Immunity and Inflammation Cochin Institute INSERM U1016 CNRS UMR8104 University of Paris Paris France

**Keywords:** Drp1, mitochondria, PD‐1, T cell, tumor‐infiltrating lymphocytes

## Abstract

Programmed cell death‐1 (PD‐1) signaling downregulates the T‐cell response, promoting an exhausted state in tumor‐infiltrating T cells, through mostly unveiled molecular mechanisms. Dynamin‐related protein‐1 (Drp1)‐dependent mitochondrial fission plays a crucial role in sustaining T‐cell motility, proliferation, survival, and glycolytic engagement. Interestingly, such processes are exactly those inhibited by PD‐1 in tumor‐infiltrating T cells. Here, we show that PD‐1^pos^ CD8^+^ T cells infiltrating an MC38 (murine adenocarcinoma)‐derived murine tumor mass have a downregulated Drp1 activity and more elongated mitochondria compared with PD‐1^neg^ counterparts. Also, PD‐1^pos^ lymphocytic elements infiltrating a human colon cancer rarely express active Drp1. Mechanistically, PD‐1 signaling directly prevents mitochondrial fragmentation following T‐cell stimulation by downregulating Drp1 phosphorylation on Ser616, via regulation of the ERK1/2 and mTOR pathways. In addition, downregulation of Drp1 activity in tumor‐infiltrating PD‐1^pos^ CD8^+^ T cells seems to be a mechanism exploited by PD‐1 signaling to reduce motility and proliferation of these cells. Overall, our data indicate that the modulation of Drp1 activity in tumor‐infiltrating T cells may become a valuable target to ameliorate the anticancer immune response in future immunotherapy approaches.

## Graphical abstract

PD‐1 signaling prevents TCR‐dependent Drp1 activation and subsequent mitochondrial fragmentation in T cells. In turn, this reduces both proliferation and migration of activated T cells. Cancer cells exploit this mechanism to downregulate T‐cell functionality within the tumor microenvironment, favoring tumor progression. These findings shed light on a new possible therapeutical approach for the treatment of solid cancers. (image made in BioRender).

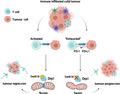

**Abbreviations**
Akt: protein kinase B
CCL19/21: CC motif chemokine ligand 19/21
DLN: draining lymph node
Drp1: dynamin‐related protein‐1
ECAR: extracellular acidification rate
ERK1/2: extracellular‐regulated kinase 1/2
ERKi: ERK1/2 inhibitor FR180204
Fis1: mitochondrial fission protein‐1
hPBT cell: human peripheral blood T cell
i.p.: intraperitoneal
IFNγ: interferon‐γ
IL‐2/7/15: interleukin‐2/7/15
MAPK: mitogen‐activated protein kinases
Mff: mitochondrial fission factor
MFI: median fluorescence intensity
Mfn1: mitofusin‐1
Mfn2: mitofusin‐2
mTOR: mammalian target of rapamycin
OCR: oxygen consumption rate
Opa‐1: optic atrophy protein‐1
OXPHOS: oxidative phosphorylation
PD‐1: programmed cell death‐1
PD‐L1: PD‐1 ligand 1
PD‐L2: PD‐1 ligand 2
PI3K: phosphatidylinositol‐3‐kinase
s.c.: subcutaneous
TCR: T‐cell receptor
T_eff_ cell: effector T cell
T_ex_ cell: exhausted T cell
TIL: tumor‐infiltrating T lymphocyte
WT: wild‐type

## 1 Introduction

Programmed cell death‐1 (PD‐1) is a T‐cell surface receptor that downregulates T‐cell activation and the immune response [1]. PD‐1 signaling is activated by PD‐1 interaction with its ligands PD‐L1 and PD‐L2, expressed on adjacent cells [2], and it dampens signals originating from T‐cell receptor (TCR) and CD28, such as the activation of mammalian target of rapamycin (mTOR) and mitogen‐activated protein kinase (MAPK) pathways [3, 4]. Besides being frequently observed in T cells during chronic infections [5], activation of PD‐1 signaling has also been widely reported in tumor‐infiltrating T cells, contributing to their functional exhaustion and poor antitumor response [6]. Consistently, antagonistic PD‐1 antibodies efficiently reinvigorate tumor‐infiltrating T cells, thereby ameliorating antitumor response [7, 8].

Mitochondria are central modulators of cellular bioenergetics, and their dynamic morphology is tightly linked to cell functions. Among the mitochondrial‐shaping proteins, dynamin‐related protein‐1 (Drp1) is the main profission protein and it is recruited from the cytosol to mitochondria thanks to post‐translational modifications and to several receptors, such as Mff and Fis1 [9, 10]. Interestingly, mitochondrial morphology is tightly linked to an optimal T‐cell functionality [11]. Particularly, Drp1‐dependent mitochondrial fragmentation sustains T‐cell motility and proliferation, and effector T (T_eff_) cell apoptosis following TCR engagement [12, 13]. Also, Drp1‐dependent mitochondrial relocation at the immunological synapse controls the influx of calcium upon T‐cell activation [14], sustaining the cMyc‐dependent upregulation of glycolytic enzymes [13, 15], thus allowing the metabolic reprogramming required to cope with the increased bioenergetic demand of an activated T cell [16, 17]. All these processes contribute to an optimal antitumor T‐cell response, which is indeed defective in T cells lacking Drp1 [13].

Of note, most of these Drp1‐dependent processes are also downregulated by PD‐1 co‐inhibitory signaling, especially when considering tumor‐infiltrating T cells. Indeed, PD‐1 signaling reduces T‐cell proliferation and motility (both of them requiring Drp1) [4, 13, 18], and it also promotes a shift from a glycolysis‐based metabolism (supported by Drp1) toward an OXPHOS‐based metabolism (requiring mitochondrial fusion) [19]. Given this striking inverse correlation, we asked whether PD‐1 signaling may modulate Drp1 activity, and to what extent this modulation may downregulate several processes in T cells. This point is of extreme importance, since the molecular mechanisms by which PD‐1 regulates the aforementioned processes in T cells are not yet completely understood.

We here show that tumor‐derived PD‐1^pos^ CD8^+^ T cells exhibit a significant downregulation of Drp1 activity and a more fused mitochondrial network. Mechanistically, PD‐1 signaling prevents Drp1 activation following T‐cell stimulation by regulating its phosphorylation on Ser616 through the modulation of extracellular‐regulated kinase 1/2 (ERK1/2) and mTOR proteins. Also, we provide evidence that Drp1 downregulation contributes to the reduced proliferation and motility of PD‐1^pos^ tumor‐infiltrating T cells, and, as a consequence, we identify Drp1 as a possible target for future therapeutic approaches aiming at restoring antitumor response in PD‐1^pos^ exhausted CD8^+^ T cells.

## 2 Materials and methods

### 2.1 Human samples

Peripheral blood samples were purified from buffy coats of healthy volunteer blood donors (independently of sex and age) under procedures approved by Institutional Review Board of Bambino Gesù Children’ Hospital (Approval of Ethical Committee No 1314/2020 prot. No 19826), including informed and written consensus for research purpose. The study methodologies conformed to the standards set by the Declaration of Helsinki. Blood cells were isolated as previously reported [12]. Briefly, cells were incubated with RosetteSep Human T‐Cell Enrichment Cocktail antibody mix (StemCell 15061). Unlabeled human peripheral blood T (hPBT) cells were isolated by density gradient over Lymphoprep (StemCell 07811), with centrifugation for 20 min at 1200rcf. Then, T cells have been collected, washed, and used for subsequent analyses. Human colon adenocarcinoma tissue sections were collected from the archives of the Tumor Immunology Laboratory, Department of Health Science according to the Helsinki Declaration and under the approval of the University of Palermo Ethical Review Board (Approval No 09/2018).

### 2.2 Mice

WT and Drp1^fl/fl^Lck:cre+ c57BL/6 mice were bred and maintained under conventional conditions at the Plaisant Srl (Castel Romano) Animal Facility. Mouse strains have been previously described [13] and are in‐house stocks. Mice were kept in cages of no more than 5‐6 mice each, divided by sex, under 12‐h/12‐h light/dark cycles, with standard temperature, humidity, and pressure conditions according to FELASA guidelines. Small red squared mouse house and paper were used for cage enrichment. Mouse health was monitored daily by veterinary staff, and health analysis for pathogens was performed every 3 months according to FELASA guidelines. All mice were sacrificed by neck dislocation at 2–3 months of age. All efforts were made to minimize animal suffering and to reduce the number of mice used, in accordance with the European Communities Council Directive of November 24, 1986 (86/609/EEC). The mouse protocol has been approved by the Allevamenti Plaisant Srl Ethical Committee as well as by the Italian Ministry of Health (Authorization #186/2020‐PR). It has been written following the ARRIVE Guidelines, and the numeric details have been chosen following the criteria described in The National Centre for the Replacement, Refinement and Reduction of Animals in Research (NC3Rs) (http://www.nc3rs.org.uk/). Sample size for the experiments performed has been established using power analysis method. Experiments involving growth of tumor cells in mice were performed using male mice.

### 2.3 Cell cultures and reagents

As previously described [12], human peripheral blood T (hPBT) cells have been cultured in RPMI 1640 medium (Thermo Fisher, Waltham, MA, USA, 21875) supplemented with 10% fetal bovine serum (Thermo Fisher 10270), 2 mm l‐glutamine (Thermo Fisher 25030081), 100 U·mL^−1^ penicillin/streptomycin (Thermo Fisher 15140130), 1× GIBCO MEM nonessential amino acids (Thermo Fisher 11140035), 1 mm sodium pyruvate (Thermo Fisher 11360039), and 100 mg·mL^−1^ gentamycin (Thermo Fisher 15750045).

Murine T cells have been isolated from mouse spleen using 70 mm Cell Strainers (Corning, NY, USA, 431751) and purified using Pan T‐Cell Isolation Kit (Miltenyi, Bergisch Gladbach, Germany, 130‐095‐130) or naïve CD8^+^ T Cell Isolation Kit (Miltenyi 130‐096‐543). Cells were cultured in the same medium used for hPBT cells (complete RPMI medium) with the only exception of 50 µm β‐mercaptoethanol (Thermo Fisher 31350‐010) addition.

MC38 tumor cells are a gift from Dr. Silvia Piconese (La Sapienza, Rome, Italy). MC38 tumor cells have been cultured in complete DMEM medium (Thermo Fisher 41966052) supplemented with 10% fetal bovine serum (Thermo Fisher 10270), 2 mm l‐glutamine (Thermo Fisher 25030081), 100 U·mL^−1^ penicillin/streptomycin (Thermo Fisher 15140130), 1× GIBCO MEM nonessential amino acids (Thermo Fisher 11140035), 1 mm sodium pyruvate (Thermo Fisher 11360039), and 50 µm β‐mercaptoethanol (Thermo Fisher 31350‐010).

Jurkat cells (in‐house stock) have been cultured in RPMI 1640 medium (Thermo Fisher 21875) supplemented with 10% fetal bovine serum (Thermo Fisher 10270), 2 mm l‐glutamine (Thermo Fisher 25030081), 100 U·mL^−1^ penicillin/streptomycin (Thermo Fisher 15140130), 1x GIBCO MEM nonessential amino acids (Thermo Fisher 11140035), and 1 mm sodium pyruvate (Thermo Fisher 11360039).

For *in vitro* activation, 2 × 10^5^ murine T cells have been stimulated with 5 µg·mL^−1^ anti‐CD3 (plate‐coated) (eBioscience, San Diego, CA, USA, 14‐0031‐86) and 1 µg·mL^−1^ anti‐CD28 (Invitrogen, Waltham, MA, USA, 14‐0281‐86) for up to 48 h in 96‐well plate. Alternatively, 5 × 10^5^ murine or human T cells have been stimulated at 1 : 1 ratio in 48‐well plate in the presence of sulfate latex 4% w/v 5 µm beads (molecular probes S37227). For each experiment, 20 × 10^6^ beads were coated on at 4 °C with 2 µg anti‐CD3 (mouse: eBioscience 14‐0031‐86; human: eBioscience 16‐0037‐85) and 1 µg anti‐CD28 (mouse: Invitrogen 14‐0281‐86; human: 16‐0289‐85) and either 7 µg of recombinant PD‐L1/B7‐H1 Fc chimera protein (mouse: R&D System 1019‐B7; human: R&D System 156‐B7) (indicated as anti‐CD3/28‐PD‐L1 beads) or 7 µg of bovine serum albumin (Sigma, St. Louis, MO, USA, A2153) (indicated as anti‐CD3/28 beads). Cells defined as unstimulated were cultured in the presence of beads coated with BSA only (on coating of 20 × 10^6^ beads with 10 µg of BSA). To modulate mTOR and ERK signaling, activated T cells have been incubated with 10 nm RAD‐001 (Novartis Oncology), 30 µm FR180204 (Tocris 3706; indicated in the figures as ERKi), 1 µm Akti‐1/2 (Tocris 5773), or 10 µm C6‐ceramide cell‐permeable ceramide analog (Enzo Life Sciences, Farmingdale, NY, USA, BML‐SL110). To inhibit autophagy, 20 µm chloroquine (Sigma C6628) has been added to cells 1 h before protein extraction.

To induce isolated murine naïve CD8^+^ T cells into an exhaustion‐like state *in vitro*, cells have been stimulated up to 4 times with 5 µg·mL^−1^ anti‐CD3 (plate‐coated) (eBioscience 14‐0031‐86) and 1 µg·mL^−1^ anti‐CD28 (Invitrogen 14‐0281‐86) for 24 h in 96‐well plate. Between each stimulation, cells have been expanded using 20 ng·mL^−1^ mouse IL‐2 (R&D System 402‐ML). Cells were considered into exhaustion‐like state after 4 stimulations (T_ex_) and were compared with effector‐like (T_eff_) cells isolated from sibling mice and stimulated only once. For *in vitro* migration experiments, T_ex_ and T_eff_ cells were finally expanded *in vitro* for additional 6 days in IL‐2‐containing medium and then used for the assays.

### 2.4 Western blot

Western blot were performed as previously described [13]. The following primary antibodies have been used: anti‐actin (Cell Signaling, Danvers, MA, USA, 4970), anti‐Drp1 (BD Bioscience 611113), anti‐pS616‐Drp1 (Cell Signaling 4494), anti‐pS637‐Drp1 (Cell Signaling 6319), anti‐Mfn2 (Abcam ab56889), anti‐Mfn1 (Santa Crux sc‐50330), anti‐Opa‐1 (BD Bioscience 612607), anti‐Fis1 (Abcam ab71498), anti‐Mff (Abcam ab129075), anti‐pT202/204‐ERK1/2 (Cell Signaling 4370), anti‐ERK1/2 (Cell Signaling 4695), anti‐Hsp90 (Cell Signaling 4877), anti‐pSer2481‐mTOR (Cell Signaling 2974), anti‐mTOR (Cell Signaling 2983), anti‐MnSOD (Enzo Life Sciences ADI‐SOD‐110), anti‐LC3B (Cell Signaling 3868), anti‐cFos (Cell Signaling 4384), anti‐GAPDH (Cell Signaling 2118), anti‐Akt1 (Cell Signaling 2938), and anti‐pThr308‐Akt (Cell Signaling 4056). All primary antibody incubations were followed by incubation with appropriated secondary HRP‐conjugated antibodies (GE Healthcare or Cell Signaling) in 5% milk plus 0.1% Tween‐20 (Sigma P2287). Detection of protein signals was performed using Clarity Western ECL substrate (Bio‐Rad 170‐5061) and Amersham Imager 600. Stripping of the membranes for reprobing has been performed using buffer containing 1% Tween‐20 (Sigma P2287), 0.1% SDS (Sigma 71729), and 0.2 M glycine (VWR M103) at pH 2.2 (two washes for 10 min).

### 2.5 Immunofluorescence

Immunofluorescence staining has been performed as previously described [12]. Anti‐TOM20 (Santa Cruz, Dallas, TX, USA, sc‐11415) primary antibody was used to identify the mitochondrial network. Nunc Lab‐Tek Chamber Slides (Thermo Fisher 154534) have been used to culture *in vitro* T cells directly on slides before fixation and were coated with 10 ng·mL^−1^ fibronectin (Millipore, Burlington, MA, USA, FC010) for 1 h at RT before adding the cells. Images were acquired using a Perkin Elmer Ultraview VoX microscope. The mitochondrial network has been always evaluated upon 0.4 mm slices z‐stack reconstructions.

### 2.6 Immunohistochemistry

Formalin‐fixed and paraffin‐embedded (FFPE) tissue samples of human colon cancer moderately differentiated (G2) cases were selected for in situ immunophenotypic analyses. Four‐micrometers‐thick sections were deparaffinized, rehydrated, and unmasked using Novocastra Epitope Retrieval Solutions pH 9 in a thermostatic bath at 98 °C for 30 min. Subsequently, the sections were brought to room temperature and washed in PBS. After neutralization of the endogenous peroxidase with 3% H_2_O_2_ and Fc blocking by a specific protein block (Leica Novocastra, Wetzlar, Germany), the samples were incubated with phospho‐DRP1 (Ser 616) (clone D9A1 Cell Signaling, 1 : 100) and PD‐1 (clone NAT105 Abcam, 1 : 50) antibodies. IHC staining was revealed using MACH 2 Double Stain 1 kit (Biocare, Pacheco, CA, USA) and DAB (3,3'‐diaminobenzidine, Leica Novocastra) and Vulcan Fast Red as substrate chromogens. Triple immunostainings were performed by incubating the same sections with CD8 antibody (clone 4B11, 1 : 50 pH9, Leica Novocastra) and anti‐mouse Alexa Fluor 488‐conjugated secondary antibody (1 : 500, Life Technologies, Carlsbad, CA, USA). Slides were analyzed under a Zeiss Axioscope A1, and microphotographs were collected using a Zeiss Axiocam 503 Color with the Zen 2.0 Software (Zeiss).

### 2.7 Flow cytometry

The following antibodies have been used to stain extracellular proteins: A488‐anti‐CD8 (BioLegend, San Diego, CA, USA, 100723), PE‐anti‐PD‐1 (eBioscience 12‐9981‐83), PECy7‐anti‐CD4 (BioLegend 100422), BV650‐anti‐CD45 (BioLegend 103151), BV421‐anti‐CD44 (BioLegend 103039), APC‐anti‐PD‐L1 (BioLegend 124311), BV421‐anti‐PD‐L2 (BioLegend 107219), PECy7‐anti‐CD8a (eBioscience 25‐0088‐42), A488‐anti‐CD4 (eBioscience 53‐0048‐42), and PE‐anti‐PD‐1 (eBioscience 12‐2799‐42). Foxp3 transcription factor staining buffer set (00‐5523‐00, eBioscience) has been used to stain intracellular proteins, detected with the following antibodies: anti‐Drp1 (BD Bioscience 611113), anti‐pS616‐Drp1 (Cell Signaling 4494), anti‐pS637‐Drp1 (Cell Signaling 6319), anti‐Mfn2 (Abcam ab56889), anti‐Mfn1 (Santa Crux sc‐50330), anti‐Opa‐1 (BD Bioscience 612607), anti‐Fis1 (Abcam ab71498), anti‐Mff (Abcam ab129075), secondary goat anti‐rabbit Alexa647 (Invitrogen A21244), secondary goat anti‐mouse Alexa647 (Jackson 115‐605‐146), and secondary goat anti‐rabbit Alexa405 (Invitrogen A31556). Primary antibodies were incubated on at 4 °C, while secondary antibodies for 1 h at RT. Background signals obtained by staining solely with secondary antibodies were subtracted from the corresponding signal from primary antibodies in the same cells to obtain MFI values reported in the figures.

To evaluate IFNγ production in tumor‐derived T cells, cells have been restimulated for 4 h in presence of 50 ng·mL^−1^ PMA (Sigma 79346), 1 μg·mL^−1^ ionomycin (Sigma I9657) and 2 μm monensin (added for the last 2 h, Sigma M5273) and then fixed and processed using Foxp3 transcription factor staining buffer set (00‐5523‐00, eBioscience) and anti‐IFNg‐PE antibody (eBioscience 12‐7311‐82). Acquisitions have been performed using BD Accuri C6 and BD FACSCelesta cytometers. Cell sorting has been performed by staining cells with the aforementioned extracellular antibodies and using BD FACS Aria III flow cytometer.

To evaluate glucose uptake, 30 μm 2‐NBDG (Thermo N13195) has been added to the cells for 20 min in DPBS. Then, cells have been washed and analyzed by flow cytometry (BD Accuri C6).

For the evaluation of the mitochondrial membrane potential, 100 nm TMRE (Thermo Fisher T669) and 100 nm MitoTrackerGreen (Thermo M7514) have been added for 20 min, and then, the cells were washed and analyzed. As a positive control for mitochondrial depolarization, cells have been pretreated with 50 μm FCCP (Sigma‐Aldrich C2920).

### 2.8 Tumor induction

Tumor inoculation was performed as previously reported [12]. Briefly, 5*10^5^ MC38 cells were injected subcutaneously into the right flank of two‐month‐old male WT or Drp1^fl/fl^ and Drp1^fl/fl^Lck:cre+ mice. Mice were kept for up to 17/18 days in animal facility, and tumor growth was monitored twice or three times per week and recorded as [longest diameter]*[shortest diameter]^2^ in cubic millimeters. At days 7, 9, 11, 14, and 16 from tumor inoculation, mice were inoculated intraperitoneal with 150 µg of InVivoMab anti‐mouse PD‐1 (CD279), clone RMP1‐14 (Bioxcell, BE0146) or 150 µg InVivoMab rat IgG2a isotype control, clone 2A3 (Bioxcell, BE0089) antibodies (in 150 µL of saline). Mice were randomly subdivided into each experimental group (a‐IgG or a‐PD‐1) before inoculation of antibodies (no specific randomization method was used). At day 17, mice were sacrificed and tumors were collected. Tumor tissues were mechanically dissociated over 70 mm‐cell strainers, and mononuclear cells were enriched from tumor‐derived cell suspensions by 40%/80% Percoll (GE Healthcare GE17‐0891‐01) density gradient, by collecting cells at the interface between 40% and 80% Percoll solution.

Isolated TILs have been used for subsequent proliferation and migration *in vitro* analyses or stained for flow cytometric measurements.

### 2.9 Seahorse analysis

Basal OCR has been measured in T cells during acute phase (unstimulated or stimulated for 12 h) or after 48 h of stimulation and 4 days of *in vitro* expansion with IL‐2, as previously described [13]. ECAR and OCR have been measured in control and Drp1‐KO T cells at 24 h and 48 h of stimulation with aCD3/28‐ and aCD3/28+PD‐L1 beads, and the relative metabolic parameters (basal glycolysis rate and basal respiration rate) were estimated as previously described [13].

### 2.10 *In vitro* proliferation, polarization, and migration assays

To evaluate the *in vitro* proliferation potential of tumor‐infiltrated CD8^+^ T cells isolated from tumor‐bearing Drp1^fl/fl^ and Drp1^fl/fl^Lck:cre+ mice, 2 × 10^5^ isolated TILs have been cultured *in vitro* in 96‐well plate in the presence of 20 ng·mL^−1^ mouse IL‐2 (R&D System 402‐ML), 20 ng·mL^−1^ mouse IL‐7 (R&D System 407‐ML), and 20 ng·mL^−1^ mouse IL‐15 (R&D System 447‐ML). The total number of cells was estimated each 2/3 days using BD Accuri C6 flow cytometer and the percentage of CD8^+^ T cells in each plate evaluated by flow cytometry staining with anti‐CD8‐A488 (BioLegend 100723) antibody.

For Transwell migration assays, 5 × 10^5^ TILs or *in vitro*‐induced T_eff_ and T_ex_ have been starved from serum for 2 h (by replacing FBS in the medium with 0.5% bovine serum albumin, Sigma A2153) and then loaded on 5 mm‐pore size Transwell filters (Costar 3421) and allowed to migrate for 2 h in the presence of 50 nm CCL19 (R&D System 440‐M3), 50 nm CCL21 (R&D System 457‐6C), or 10% fetal bovine serum (Thermo Fisher 10270).

For the polarization assay, 2 × 10^5^ cells have been starved from serum for 2 h (by replacing FBS in the medium with 0.5% bovine serum albumin, Sigma A2153). Then, cells were allowed to adhere to 10 mg·mL^−1^ fibronectin‐coated (Millipore FC010) microscope slides (Thermo Fisher ER302W‐CE24) for 30 min and stimulated by adding 50 nm CCL19 (R&D System 440‐M3) and 50 nm CCL21 (R&D System 457‐6C) for 15 min before fixation and immunostaining. Alternatively, after serum starvation, 2 × 10^6^ cells were kept in 1.5‐mL Eppendorf tube and stimulated by adding 50/200 nm CCL19 (R&D System 440‐M3) and 50/200 nm CCL21 (R&D System 457‐6C) for 15 min before directly proceeding to protein extraction. When indicated, cells were preincubated for 2 h with 5 µg·mL^−1^ of BSA or PD‐L1 (for immunofluorescence) or BSA‐ and PD‐L1‐coated beads (10 µg for 20 mln beads) (For western blot).

### 2.11 Electroporation

Jurkat cells have been electroporated using Neon Transfection System (Thermo Fisher) following manufacturer instructions and kept on in antibiotic‐free medium before being washed and used for protein extraction. The following siRNA has been used: siERK1/2 (Cell Signaling 6560), siCTRL (Santa Cruz sc‐37007), and simTOR (Cell Signaling 6381).

### 2.12 Statistical analysis

**Fig. 1:**
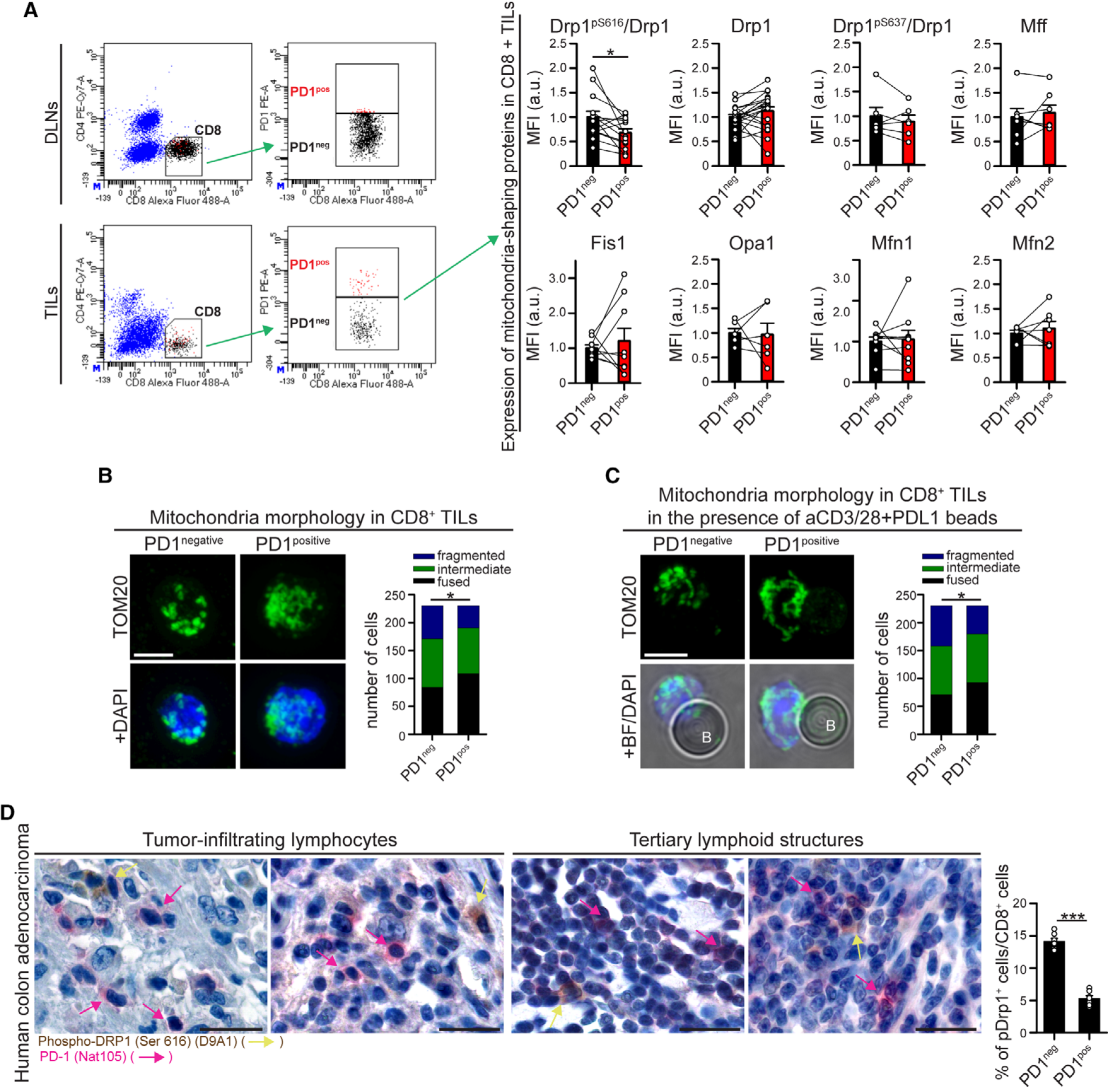
PD‐1^positive^ and PD‐1^negative^ CD8^+^ T cells within MC38‐derived tumor microenvironment show different mitochondrial morphologies. (A) Draining lymph node (DLN)‐derived T cells and tumor‐infiltrating lymphocytes (TILs) have been isolated from 18‐day‐old MC38‐derived tumor mass grown in WT c57BL/6 mice, and the expression of the indicated mitochondrial‐shaping proteins has been evaluated into PD‐1^negative^ (PD‐1^neg^) and PD‐1^positive^ (PD‐1^pos^) CD8^+^ T‐cell subpopulations. Representative gating strategy to distinguish PD‐1^neg^ and PD‐1^pos^ CD8^+^ T cells is shown on the left. Graphs on the right indicate the normalized median fluorescence intensity (MFI) of the indicated proteins evaluated by intracellular flow cytometry in PD‐1^neg^ and PD‐1^pos^ CD8^+^ T cells from the same mice and expressed as normalized intensity ratio relative to secondary antibodies alone (pS616‐Drp1 *n* = 14; Drp1 *n* = 20; Mfn1 and Fis1 *n* = 9; Mfn2 and Mff *n* = 7; Opa‐1 and pS637‐Drp1 *n* = 6; paired *t*‐tests). (B‐C) PD‐1^negative^ (PD‐1^neg^) and PD‐1^positive^ (PD‐1^pos^) CD44^+^ CD45^+^ CD8^+^ T cells have been sorted and purified form 18‐day‐old MC38‐derived tumor mass grown in WT c57BL/6 mice. Gating strategy is shown in Fig. S1. Mitochondrial morphology was evaluated by immunofluorescence (anti‐TOM20 staining) and upon z‐stack reconstruction. In (B), cells have been fixed immediately after purification and processed for immunostaining. In (C), cells have been stimulated for 2 h in the presence of beads coated with aCD3/28 Abs plus PD‐L1 and then fixed and processed for immunostaining. For each panel, representative images of the observed mitochondrial morphologies are shown on the left, while graphs on the right show the distribution of cells into the indicated category according to mitochondrial morphology in PD‐1^neg^ and PD‐1^pos^ CD8^+^ T cells (*n* = 230 cells each condition from 8 [B, unstimulated] or 10 [C, stimulated with beads] pooled mice; chi‐square tests). (D) Representative microphotographs of double‐marker immunohistochemistry for PD‐1 (rose; rose arrows) and Drp1‐pSer616 (brown; yellow arrows) expression in lymphoid elements infiltrating human colon cancer. The graph on the right indicates the percentage of pDrp1^pos^ cells among all PD‐1^neg^ and PD‐1^pos^ CD8^+^ T cells (*n* = 7 patients, see Fig. S1G for the triple immunostaining). Data are shown as mean ± SEM. Scale bar: 5 µm in B and C and 50 µm in D. Significance is indicated as follows: *= *P* < 0.05; ***= *P* < 0.001. Statistical tests used: paired *t*‐test (A); chi‐square test (B‐C); unpaired *t*‐test (D).

In the Figure legends, ‘*n*’ indicates either the number of independent experiments (*in vitro* primary cells) or the number of mice used. Data are expressed as mean ± SEM from at least three independent experiments unless specified otherwise (Microsoft Office Excel and SigmaPlot v12.5 have been used for analysis). The number of mice used has been estimated using the power analysis method. All the acquisitions of the experiments have been performed blinded without knowing the specific conditions of each sample. Comparisons between groups were done using two‐tailed Student’s *t*‐test (two groups) or one‐way and two‐way ANOVA (multiple groups and repeated measurements, adjustments for pairwise comparisons were performed using Holm–Sidak method). Mann–Whitney rank‐sum test or ANOVA on ranks has been used if samples did not meet assumptions of normality and/or equal variance. Chi‐square test has been used to evaluate data in Figure 1B. *P*‐values are indicated in the Figures as follows: * = *P* < 0.05, ** = *P* < 0.01, *** = *P* < 0.001.

## 3 Results

### 3.1 PD‐1^pos^ CD8^+^ T cells from MC38‐derived murine tumors show a reduced mitochondrial fission

To investigate whether PD‐1 signaling modulates the morphology of the mitochondrial network in tumor‐infiltrating T lymphocytes (TILs), we looked at PD‐1^neg^ and PD‐1^pos^ CD8^+^ T cells infiltrating an 18‐day‐old solid tumor mass derived from s.c. inoculation of MC38 cells (murine adenocarcinoma) in c57BL/6 WT mice. We took advantage of this tumor model, since it is characterized by (a) a high level of T‐cell infiltration [13], (b) the presence of PD‐1^pos^ CD8^+^ (but largely not CD4^+^) TILs (Fig. S1A), (c) the expression of PD‐1 ligand PD‐L1 (but not PD‐L2) in tumor cells (Fig. S1B), and (d) the expression of PD‐L1 and PD‐L2 in ca. 10% and 2% of tumor‐infiltrating CD45^pos^ non‐T (i.e., CD4^neg^ CD8^neg^) cells (Fig. S1C). By comparing the expression levels of different mitochondrial‐shaping proteins in CD8+ TILs, we observed that PD‐1^pos^ CD8^+^ TILs do not show altered levels of total Drp1 compared with PD‐1^neg^ counterparts (Fig. 1A). However, we observed a specific downregulation of Drp1 phosphorylation on its activating residue Ser616 (Fig. 1A and Fig. S1D), while the inhibitory phosphorylation on Ser637 did not vary. Also, we did not observe any differences in the expression of other main profusion (Mfn1, Mfn2, Opa‐1) or profission (Fis1, Mff) proteins (Fig. 1A).

Consistent with their reduced level of active Drp1, PD‐1^pos^ CD8^+^ TILs show an altered morphology of mitochondria. Indeed, while PD‐1^neg^ CD44^+^ CD8^+^ TILs show a fragmented network (as expected from activated T cells), mitochondria in PD‐1^pos^ CD44^+^ CD8^+^ TILs are more fusion‐prone (Fig. 1B, Fig. S1E). Interestingly, this was observed also in sorted PD‐1^pos^ CD8^+^ TILs stimulated for 2 h (a time window not sufficient to upregulate PD‐1 expression in PD‐1^neg^ CD44^+^ CD8^+^ TILs, Fig. S1F) with beads coated with anti‐CD3/28 antibodies plus PD‐L1 (PD‐1 ligand) before fixation (Fig. 1C). This suggests that such an altered mitochondrial morphology in PD‐1^pos^ CD8^+^ TILs may also influence T‐cell activation upon antigen encounter in a PD‐L1‐rich microenvironment (such as a tumor mass).

Last, we extended these observations to a homologue human tumor context, by staining moderately differentiated (G2) human colon carcinoma sections with anti‐CD8, anti‐PD‐1, and anti‐Drp1‐pSer616 antibodies. Of note, we observed a reduced percentage of phospho‐Drp1^pos^ elements in PD‐1^pos^ CD8^+^ T cells compared with PD‐1^neg^ CD8^+^ T cells (Fig. 1D and Fig. S1G). This suggests that also in a human tumor context, PD‐1^pos^ CD8^+^ T cells have a downregulated Drp1 activity.

In sum, tumor‐infiltrating PD‐1^pos^ CD8^+^ T cells show a tendency toward a more interconnected mitochondrial morphology, associated with a reduced activation of Drp1.

### 3.2 PD‐1 signaling prevents Drp1 activation and mitochondrial fragmentation in both murine and human T cells, upon *in vitro* stimulation

**Fig. 2:**
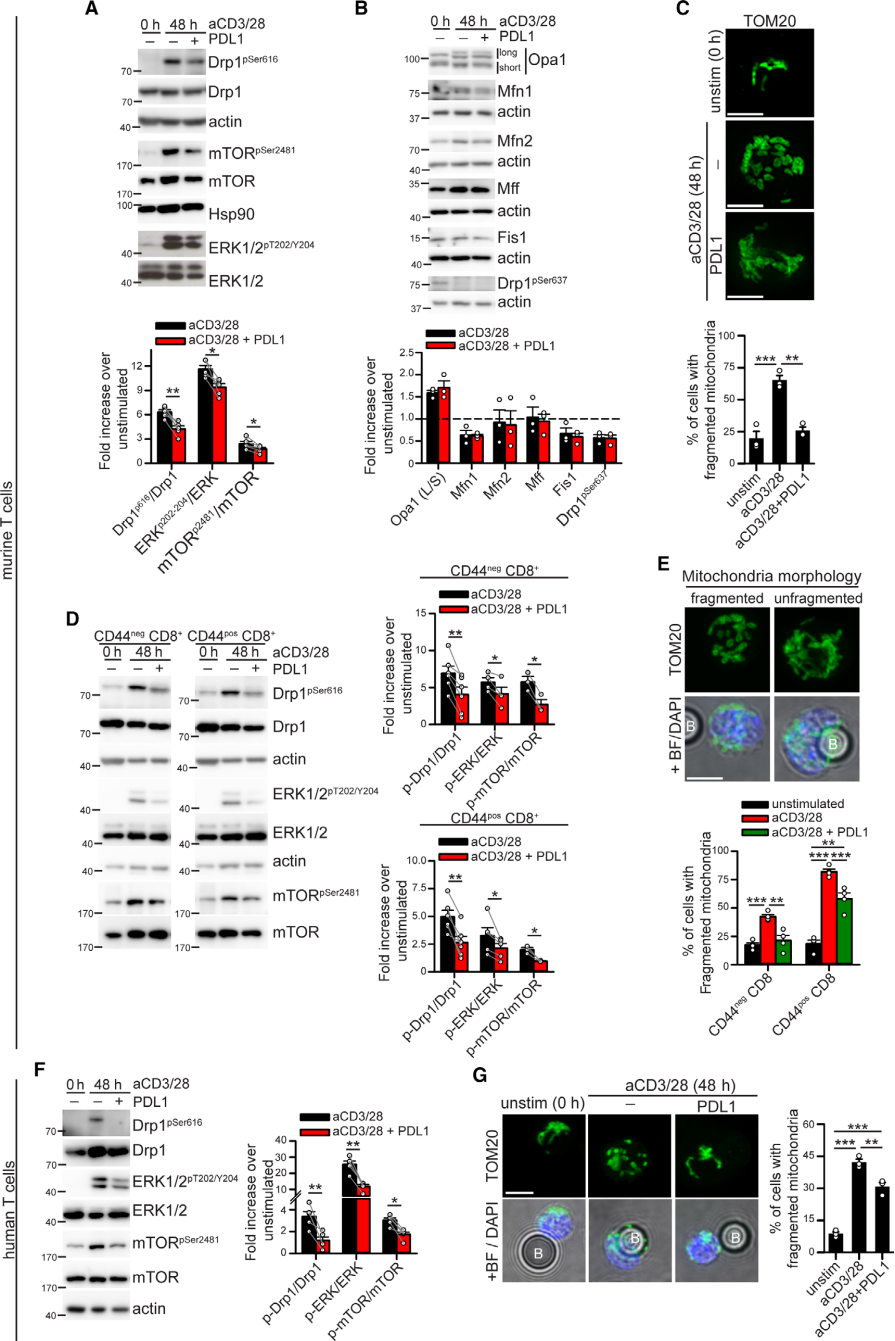
PD‐1 signaling downregulates Drp1‐dependent mitochondrial fragmentation in murine and human T cells. (A‐B) Murine T cells have been isolated from spleen of WT c57BL/6 mice and left unstimulated (0 h) or stimulated for 48 h with anti‐CD3/28‐ or anti‐CD3/28‐PD‐L1 beads. The expression level of the indicated (phospho)‐proteins has been evaluated by western blot (A *n* = 5, paired *t*‐tests; B *n* = 3). (C) Representative immunofluorescence images showing the mitochondrial network (anti‐TOM20 staining) in murine T cells isolated and stimulated as in (A). Quantification of the percentage of cells showing fragmented mitochondria in each condition is reported in the graph below (*n* = 3). (D) Murine CD44^neg^ (naïve) and CD44^pos^ (antigen experienced) CD8^+^ T cells have been isolated from spleen of WT c57BL/6 mice and left unstimulated (0 h) or stimulated for 48 h with anti‐CD3/28‐ or anti‐CD3/28‐PD‐L1 beads. The expression level of the indicated (phospho)‐proteins has been evaluated by western blot in both CD8^+^ T‐cell subsets (pDrp1 *n* = 6; pERK naive *n* = 4; pERK memory *n* = 5; p‐mTOR *n* = 3; paired *t*‐tests). (E) Immunofluorescence images showing representative mitochondrial morphologies (anti‐TOM20 staining; ‘B’ indicates a bead) observed in CD44^neg^ and CD44^pos^ CD8^+^ T cells isolated and stimulated as in (E). Quantification of the percentage of cells showing fragmented mitochondria in each condition is reported in the graph below (*n* = 4). (F) Human T cells have been isolated from peripheral blood and left unstimulated (0 h) or stimulated for 48 h with anti‐CD3/28‐ or anti‐CD3/28‐PD‐L1 beads. The expression level of the indicated (phospho)‐proteins has been evaluated by western blot (*n* = 5; paired *t*‐tests). (G) Representative immunofluorescence images showing the mitochondrial network (anti‐TOM20 staining; ‘B’ indicates a bead) in human T cells isolated and stimulated as in (F). Quantification of the percentage of cells showing fragmented mitochondria in each condition is reported in the graph on the right (*n* = 3). Data are shown as mean ± SEM. Scale bar: 5 µm in C, E and G. Significance is indicated as follows: *= *P* < 0.05; **= *P* < 0.01; ***= *P* < 0.001. Statistical tests used: paired *t*‐test (A, D, F); unpaired *t*‐test (B), one‐way ANOVA with Holm–Sidak *post* *hoc* (C, E, G).

To investigate whether such an altered Drp1 expression in PD‐1^pos^ CD8^+^ TILs is directly caused by PD‐1 activation, we switched to an *in vitro* system to specifically modulate PD‐1 signaling. To this aim, we stimulated *in vitro* T cells isolated from spleen of WT mice with beads coated with anti‐CD3/28 plus BSA (thereafter aCD3/28 beads) or anti‐CD3/28 antibodies plus PD‐L1 (thereafter aCD3/28‐PD‐L1 beads) for 48 h. Of note, T‐cell stimulation with aCD3/28 beads leads to the activation of mTOR, ERK1/2, and Drp1 by phosphorylation (Fig. 2A) and to the upregulation of PD‐1 surface expression (Fig. S2A). As expected, concomitant activation of PD‐L1/PD‐1 axis during T‐cell stimulation dampens activation of both mTOR and ERK1/2 (Fig. 2A) [3, 4]. Interestingly, we also observed that Drp1 phosphorylation on Ser616 is strongly reduced by the engagement of PD‐1 signaling (Fig. 2A), while no significant difference was observed for other mitochondrial‐shaping proteins (Fig. 2B). In line with this, while T cells engage the fragmentation of mitochondria upon activation [12, 13, 20], in the presence of PD‐L1 the same cell type retains a mitochondrial morphology more similar to unstimulated counterparts (Fig. 2C). Of note, mitochondria of murine activated T cells might characteristically appear slightly swollen when fragmented (Fig. 2C), still being fully functional (Fig. S2B, C). In addition, such an absence of fragmented mitochondria in PD‐L1‐stimulated T cells is not due to ongoing mitophagy (which could hypothetically promote the clearance of the small and fragmented mitochondria) (Fig. S2D).

Next, we asked whether such PD‐1‐dependent regulation of Drp1 is restricted or not to some specific T‐cell subpopulation. Therefore, we looked more closely at naïve (CD44^neg^) and antigen experienced (CD44^pos^) CD8^+^ T‐cell subpopulations, which, once activated, may undergo exhaustion within the tumor microenvironment. Of note, although naïve CD8^+^ T cells do not express PD‐1 on their cell surface, they rapidly acquire PD‐1 expression as early as 12 h after stimulation with aCD3/28 beads (as also observed for naïve CD4^+^ T cells), and the concomitant presence of PD‐L1 ligand does not affect such upregulation (Fig. S2E). We thus isolated both subpopulations from the spleen of WT mice and stimulated them *in vitro* with aCD3/28‐ or aCD3/28‐PD‐L1 beads for 48 h, which is the optimal time point to detect Drp1 phosphorylation, especially in naïve CD8^+^ T cells (Fig. S2F). Interestingly, we found that engagement of PD‐1 signaling during cell activation dampens Drp1 phosphorylation and mitochondrial fragmentation in both CD44^pos^ and CD44^neg^ CD8^+^ subsets (Fig. 2D, E), in parallel with a reduced activation of ERK and mTOR pathways (Fig. 2D).

Last, a PD‐1‐dependent Drp1 modulation was also observed in human T cells isolated from healthy donors’ peripheral blood (hPBT). Similar to their murine counterpart, hPBT cells stimulated for 48 h with aCD3/28‐PD‐L1 beads show a significantly lower Drp1 phosphorylation on Ser616 (Fig. 2F) and a reduced percentage of cells with fragmented mitochondria (Fig. 2G) than aCD3/28‐stimulated hPBTs, which also display higher mTOR and ERK1/2 activation (Fig. 2F).

Overall, these data indicate that the engagement of PD‐1 signaling during T‐cell activation prevents Drp1 phosphorylation and mitochondrial fragmentation both in mice and in humans.

### 3.3 PD‐1 signaling downregulates Drp1 activation by modulating mTOR and ERK pathways

**Fig. 3:**
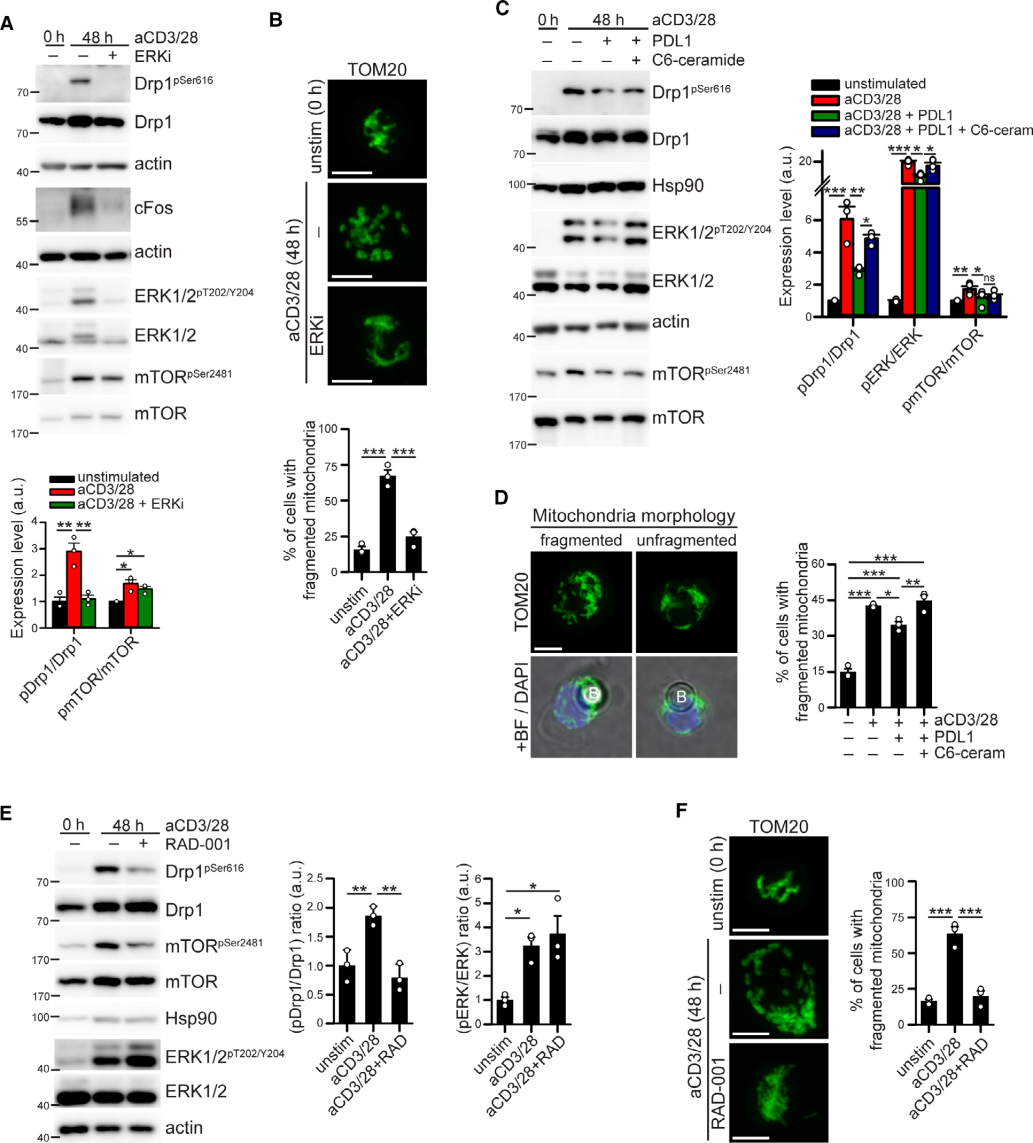
PD‐1 signaling downmodulates Drp1 activity via ERK and mTOR pathways. (A‐B) Murine T cells have been isolated from spleen of WT c57BL/6 mice and stimulated for 48 h with anti‐CD3/28‐coated beads in the presence or not of 30 µm FR180204 (ERK inhibitor: ERKi). In (A) is reported the expression level of the indicated (phospho)‐proteins evaluated by western blot and quantified in the graph below (*n* = 3). In (B) are reported representative immunofluorescence images showing the mitochondrial network (anti‐TOM20 staining) in murine T cells. Quantification of the percentage of cells showing fragmented mitochondria in each condition is reported in the graph below (*n* = 3). (C‐D) hPBT cells have been isolated from spleen of WT c57BL/6 mice and stimulated for 48 h with anti‐CD3/28‐ or anti‐CD3/28‐PD‐L1 beads in the presence or not of 10 µm C6‐ceramide (ERK activator). In (C) is reported the expression level of the indicated (phospho)‐proteins evaluated by western blot and quantified in the graph below (*n* = 3). In (D) are reported representative immunofluorescence images showing the mitochondrial network (anti‐TOM20 staining, ‘B’ indicates a bead) in hPBT cells. Quantification of the percentage of cells showing fragmented mitochondria in each condition is reported in the graph below (*n* = 3). (E–F) Murine T cells have been isolated from spleen of WT c57BL/6 mice and stimulated for 48 h with anti‐CD3/28‐coated beads in the presence or not of 10 nm RAD‐001 (mTOR inhibitor). In (E) is reported the expression level of the indicated (phospho)‐proteins evaluated by western blot and quantified in the graphs on the right (*n* = 3). In (F) are reported representative immunofluorescence images showing the mitochondrial network (anti‐TOM20 staining) in murine T cells. Quantification of the percentage of cells showing fragmented mitochondria in each condition is reported in the graph on the right (*n* = 3). Data are shown as mean ± SEM. Scale bar: 5 µm in B, D and F. Significance is indicated as follows: *= *P* < 0.05; **= *P* < 0.01; ***= *P* < 0.001. Statistical tests used: one‐way ANOVA with Holm–Sidak *post hoc* (A, B, D, E, F); one‐way ANOVA repeated measurements with Holm–Sidak *post hoc* (C).

Next, we aimed at better investigating the molecular pathways linking PD‐1 activation to the downregulation of Drp1 activity. It is well established that PD‐1 dampens the signaling pathways originating from TCR and CD28 activation [3, 4], such as PI3K/Akt/mTOR [3] and MAPK/ERK pathway [4], as indeed confirmed by our results (Fig. 2A, D, F). Since both these pathways have been reported to modulate Drp1‐dependent mitochondrial fragmentation [21, 22], we tested whether their inhibition is sufficient to reduce Drp1 activation upon T‐cell stimulation. Of note, ERK1/2 inhibitor FR180204 (ERKi) prevents both Drp1 phosphorylation and mitochondrial fragmentation in activated T cells (Fig. 3A, B), this without affecting mTOR phosphorylation (Fig. 3A). Further, we rescued ERK1/2 activity downstream of PD‐L1/PD‐1 engagement by using low doses of C6‐ceramide (to avoid apoptosis induction), a known activator of the ERK pathway [23]. Ceramide, which slightly rescues ERK phosphorylation without affecting that of mTOR (Fig. 3C), also rescues Drp1 phosphorylation and mitochondrial fragmentation in PD‐1‐engaged hPBT cells during activation (Fig. 3C, D). Next, also the mTOR inhibitor RAD‐001 [24] prevents both Drp1 phosphorylation and mitochondrial fragmentation during T‐cell activation (Fig. 3E, F), without affecting ERK phosphorylation (Fig. 3E). Furthermore, we confirmed also in Jurkat cells that the specific silencing of both ERK1/2 and mTOR by siRNA downregulates Drp1 phosphorylation on Ser616 (Fig. S3A). In addition, Drp1 phosphorylation upon T‐cell activation is reduced also when inhibiting the activity of Akt, the kinase upstream of mTOR (Fig. S3B). Interestingly, while ERK1/2 is known to directly phosphorylate Drp1 on Ser616 [21], it is currently unknown how mTOR may regulate Drp1 activity. An extensive crosstalk between mTOR and ERK pathways has been frequently reported [25, 26]. Therefore, mTOR may regulate Drp1 in T cells via the modulation of ERK1/2. However, we here observed that a low dose (10 nm) of mTOR inhibitor is sufficient to prevent Drp1 phosphorylation without affecting ERK1/2 activity (Fig. 3E). In line with this, pharmacological inhibition of Akt prevents both mTOR and Drp1 phosphorylation without affecting ERK1/2 upon T‐cell activation (Fig. S3B). Therefore, at least in T cells, mTOR seems to regulate Drp1 in a ERK1/2‐independent way.

In sum, the activation of PD‐1 signaling during T‐cell stimulation reduces Drp1 phosphorylation and mitochondrial fragmentation presumably through an inhibition of both ERK and mTOR pathways downstream of TCR/CD28 signaling.

Recently, an *in vitro* protocol based upon subsequent cycles of CD3/CD28 stimulation (see Methods for details) has been developed to induce a TCR/CD28‐hyporesponsive state in CD8^+^ T cells, thus mimicking an exhausted‐like condition of diminished proliferative and cytotoxic potential without the need of a concurrent engagement of inhibitory coreceptors, such as PD‐1 [27]. Therefore, we decided to take advantage of this protocol to understand whether Drp1 could be modulated also in this context. To this aim, we compared Drp1 phosphorylation and mitochondrial morphology in TCR/CD28 responder T_eff_‐like cells (i.e., stimulated up to two times with aCD3/CD28 Abs) and nonresponder T_ex_‐like cells (i.e., the same cells stimulated three or more times). Interestingly, we observed that Drp1 phosphorylation and fragmentation of mitochondria are strongly reduced in T_ex_‐like cells compared with T_eff_‐like ones (Fig. S3C, D).

Collectively, these data argue that Drp1 downregulation could be a common feature observed during the T‐cell exhaustion induced either by the activation of inhibitory coreceptors (such as PD‐1) or by a chronic hyporesponsive state due to TCR unresponsiveness.

### 3.4 Drp1 is required for an efficient reduction of tumor growth mediated by anti‐PD‐1 therapy

**Fig. 4:**
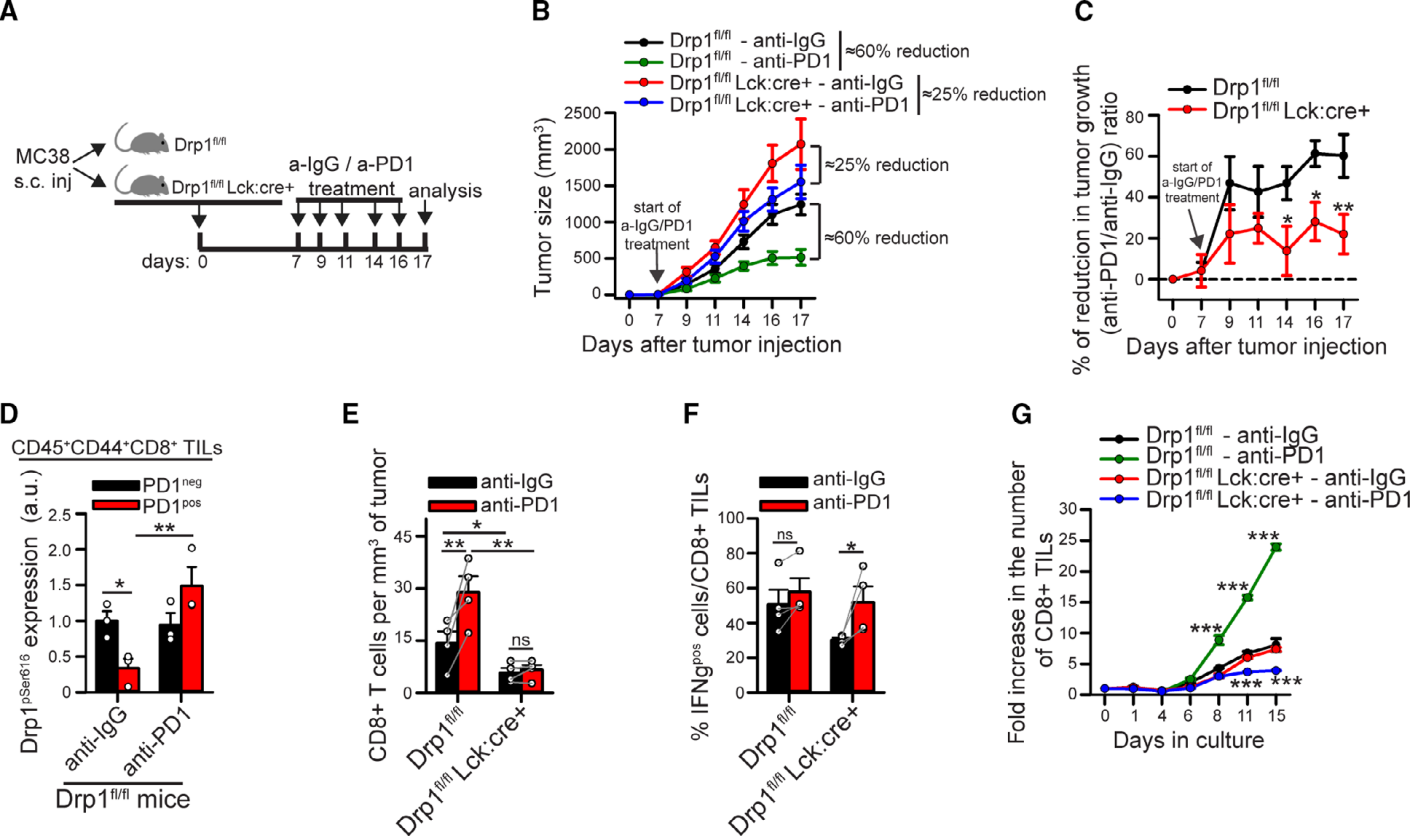
The downregulation of Drp1 activity in tumor‐derived PD‐1^positive^ T cells contributes to tumor growth. (A) Schematic representation of the experimental plan. (B) Size of MC38‐derived tumors grown for the indicated days in control Drp1^fl/fl^ or conditional‐KO Drp1^fl/fl^Lck:cre+ mice inoculated with anti‐IgG or anti‐PD‐1 antibodies as indicated in (A) (Drp1^fl/fl^ anti‐IgG *n* = 8; Drp1^fl/fl^ anti‐PD‐1 and Drp1^fl/fl^Lck:cre+ anti‐IgG *n* = 12; Drp1^fl/fl^Lck:cre+ anti‐PD‐1 *n* = 14). (C) Relative percentage of the reduction in tumor growth (anti‐PD‐1 / anti‐IgG ratio) calculated from data in (B) for control Drp1^fl/fl^ or conditional‐KO Drp1^fl/fl^Lck:cre+ mice (Drp1^fl/fl^ *n* = 12; Drp1^fl/fl^Lck:cre+ *n* = 14). (D) Quantification of the Drp1‐pSer616 median fluorescence intensity (MFI) evaluated by intracellular flow cytometry in CD45^+^ CD44^+^ CD8^+^ PD‐1^neg^ and PD‐1^pos^ TILs from control Drp1^fl/fl^ mice. The MFI value obtained in corresponding TILs from conditional‐KO Drp1^fl/fl^Lck:cre+ mice has been used as negative control and subtracted from the corresponding population in Drp1^fl/fl^ mice to obtain pSer616‐Drp1 MFI data reported in the graph (*n* = 3). (E) Absolute number of CD8^+^ T cells per mm^3^ of tumor collected from MC38‐derived tumor masses grown for 17 days as described in (A) (*n* = 4; two‐way ANOVA on repeated measurements). (F) TILs have been isolated from MC38‐derived tumor masses grown for 17 days as described in (A) and stimulated *in vitro* for 4 h to evaluate IFNγ production. Percentage of IFNγ^pos^ cells among CD8^+^ T cells in each condition is reported in the graph (*n* = 4; two‐way ANOVA on repeated measurements). (G) TILs have been isolated from MC38‐derived tumor masses grown for 17 days as described in (A) and cultured *in vitro* for the indicated days in the presence of IL‐2, IL‐7, and IL‐15 cytokines. Quantification of the fold increase in the absolute number of CD8^+^ T cells per day (starting value at day 0 = 1) is reported in the graph. Significance is indicated for each single point only if that day the population differs statistically from all the other 3 populations (*n* = 3). Data are shown as mean ± SEM. Significance is indicated as follows: *=*P* < 0.05; **=*P* < 0.01; ***=*P* < 0.001. Statistical tests used: two‐way ANOVA with Holm–Sidak *post hoc* (C, D, G); two‐way ANOVA repeated measurements with Holm–Sidak *post hoc* (E, F).

Given the importance of Drp1 in regulating multiple processes in T cells, we asked whether the ability of anti‐PD‐1 therapy to reduce solid tumor growth requires the restoration of Drp1 activity in tumor‐infiltrating PD‐1^pos^ CD8^+^ T cells. To answer this point, we analyzed s.c. MC38‐derived tumors in mice, whose growth is significantly reduced by (a) treatments with antagonistic anti‐PD‐1 Abs [28] and (b) by a functional Drp1 in T cells [13]. We inoculated s.c. MC38 cells into both control (Drp1^fl/fl^) and Drp1 conditional‐KO mice (Drp1^fl/fl^Lck:cre+, T‐cell‐restricted Drp1 ablation, indicated as Drp1‐cKO) (Fig. S4A), which we previously characterized [13]. After one week, we treated these mice with either anti‐IgG (control) or antagonistic anti‐PD‐1 antibody every 2/3 days for up to 10 days (Fig. 4A). Of note, we found that anti‐PD‐1 treatment is much more effective in reducing tumor growth in control mice than in mice where Drp1 was absent in T cells (Drp1‐cKO) (Fig. 4B). Even when correcting for the larger tumor volume in Drp1‐cKO mice compared with control mice [13], we still observed a from 60% to ca. 20% in the efficacy of anti‐PD‐1 therapy in Drp1‐cKO mice (Fig. 4C). Of note, Drp1 ablation in T cells does not affect *per se* PD‐1 expression following T‐cell activation (Fig. S4B), thus excluding that the impaired effect of anti‐PD‐1 therapy in Drp1‐cKO mice can be due to an altered expression of PD‐1. In addition, we found that the strong reduction in tumor growth by anti‐PD‐1 treatment in control (Drp1^fl/fl^) mice correlates with a rescue of Drp1 phosphorylation in PD‐1^pos^ CD8^+^ T cells to a level comparable to PD‐1^neg^ CD8^+^ T cells (Fig. 4D). On the contrary, in Drp1‐cKO mice (where Drp1 is absent and therefore cannot be ‘rescued’), anti‐PD‐1 treatment is much less efficient in reducing tumor growth. Overall, these data suggest that the restoration of Drp1 activity in PD‐1^pos^ CD8^+^ TILs is a key step for the effectiveness of the anti‐PD‐1 therapy.

Next, we tried to get more insight into the cellular processes requiring the rescue of Drp1 activity during anti‐PD‐1 therapy. Of note, while anti‐PD‐1 treatment increases the number of CD8^+^ TILs recovered from the tumor mass (per mm^3^), this effect is not observed in Drp1‐cKO mice (Fig. 4E), indicating that Drp1 is required to mediate such anti‐PD‐1‐dependent increase in CD8^+^ TILs accumulation. On the contrary, PD‐1‐dependent regulation of IFNγ production does not involve Drp1 (Fig. 4F). We previously reported that Drp1 is also required to sustain T‐cell clonal expansion after stimulation, both *in vitro* and *in vivo* [13]. Therefore, we asked whether the inability of Drp1‐KO CD8^+^ TILs to increase their number within the tumor mass upon anti‐PD‐1 treatment may depend or not on their impaired proliferation. To this aim, we isolated TILs from MC38‐derived tumor masses grown in control or Drp1‐cKO mice (treated with anti‐IgG or anti‐PD‐1) and let them expand *in vitro* in the presence of IL‐2, IL‐7 and IL‐15 cytokines. Interestingly, while the anti‐PD‐1 treatment significantly increases the fold expansion of control CD8^+^ T cells, this effect is completely lost in Drp1‐KO CD8^+^ T cells (Fig. 4G). These data suggest that Drp1 inhibition may be at least one of the mechanisms by which PD‐1 signaling reduces the proliferation of PD‐1^pos^ CD8^+^ TILs.

In sum, a functional Drp1 is required for the efficacy of the anti‐PD‐1 treatment in reducing MC38‐derived tumor growth in mice. Also, PD‐1 signaling may mediate the reduction in CD8^+^ TILs proliferative potential via Drp1 downregulation.

### 3.5 Reduced motility of PD‐1^pos^ and *in vitro* exhausted‐like T cells correlates with altered Drp1‐dependent mitochondrial remodeling

**Fig. 5:**
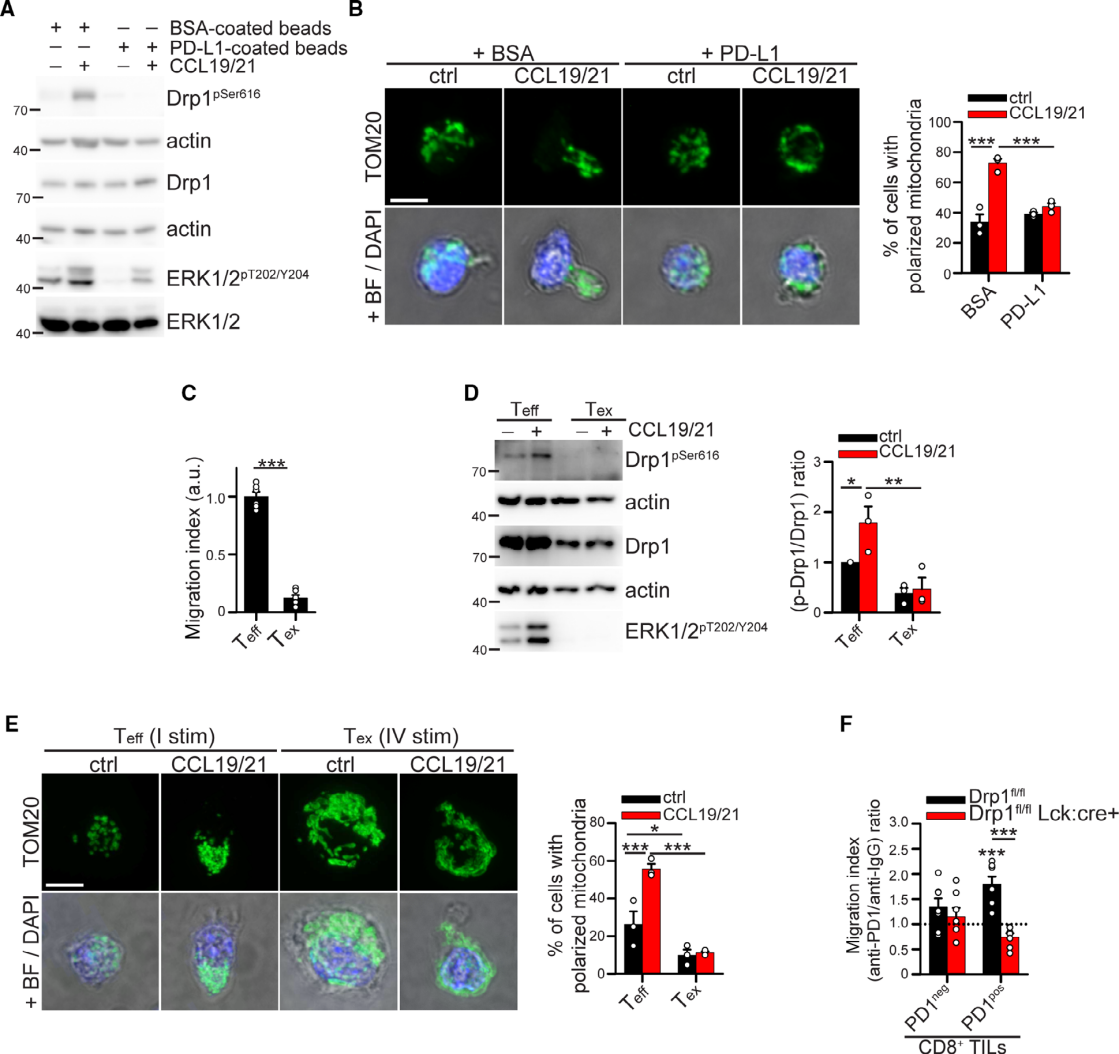
The downregulation of Drp1 activity contributes to the reduced motility of PD‐1^positive^ T cells. (A‐B) Murine T cells have been stimulated 48 h with plate‐coated anti‐CD3 (5 µg·mL^−1^) and soluble anti‐CD28 (1 µg·mL^−1^) and then expanded for 3 days with IL‐2. In (A), cells have been incubated for 2 h with BSA‐ or PD‐L1‐coated beads (10 µg for 20 mln beads), and then, 200 nm CCL19 and CCL21 have been added for 20 min, proteins have been extracted, and the expression level of the indicated (phospho)‐proteins has been evaluated by western blot (A, *n* = 1 experiment representative of three independent experiments). In (B), cells have been left to adhere for 30 min to fibronectin‐coated slides and were incubated with either 5 µg·mL^−1^ BSA or PD‐L1 for 2 h. Then, 200 nm CCL19 and CCL21 have been added for 20 min, and cells were fixed and processed for immunostaining to analyze mitochondrial morphology by immunofluorescence (TOM20, green) (B, *n* = 3). (C‐E) Murine exhausted T cells (Tex) have been generated *in vitro* through 4 cycles of aCD3/28‐mediated stimulation (24 h) and IL‐2‐mediated expansion (6 days) and compared to effector T cells (T_eff_) generated through a single cycle of stimulation and expansion. After the last 6 days in IL‐2‐containing medium, cells have been starved from serum for 2 h, and then, the following assays were performed. In (C), the migration index in response to 50 nm CCL19/CCL21 gradient for 2 h has been calculated using Transwell migration assay (*n* = 6). In (D), the cells have been stimulated with 50 nm CCL19 and 50 nm CCL21 chemokines for 15 min in an Eppendorf tube, and then, proteins were extracted. The expression level of the indicated (phospho)‐proteins has been evaluated by western blot (*n* = 3). In (E), cells have been left to adhere for 30 min to fibronectin‐coated slides. Then, cells have been stimulated with 50 nm CCL19 and 50 nm CCL21 chemokines for 15 min and then fixed and processed for immunostaining. Representative images showing the mitochondrial network (anti‐TOM20 staining) in effector T (T_eff_) and exhausted T (T_ex_) cells are shown on the left. Quantification of the percentage of cells showing fragmented mitochondria in each condition is reported in the graph on the right (*n* = 3). (F) TILs have been isolated from MC38‐derived tumor masses grown for 17 days in control Drp1^fl/fl^ or conditional‐KO Drp1^fl/fl^Lck:cre+ mice inoculated with anti‐IgG or anti‐PD‐1 antibodies as indicated in Fig. 5A. Then, TILs were starved from serum for 2 h and allowed to migrate in response to 10% fetal bovine serum for 2 h using Transwell migration assay. The graph indicates the relative (anti‐PD‐1 / anti‐IgG) migration index calculated for PD‐1^neg^ and PD‐1^pos^ CD8^+^ TILs isolated from tumor‐bearing control Drp1^fl/fl^ or conditional‐KO Drp1^fl/fl^Lck:cre+ mice inoculated with anti‐IgG or anti‐PD‐1 antibodies as indicated in Fig. 4A (*n* = 7). Data are shown as mean ± SEM. Scale bar: 5 µm in B and E. Significance is indicated as follows: *=*P* < 0.05; **=*P* < 0.01; ***=*P* < 0.001. Statistical tests used: two‐way ANOVA with Holm–Sidak *post hoc* (B, D, E); unpaired *t*‐test (C), one‐way ANOVA with Holm–Sidak *post hoc* (F).

Besides controlling T‐cell proliferation, Drp1 is required to sustain T‐cell motility by favoring mitochondrial repositioning at the uropod, and it is directly phosphorylated on Ser616 in response to chemokine stimulation [13]. Since T‐cell motility is another process dampened by PD‐1 signaling [18], we asked whether PD‐1 signaling may exploit the downregulation of Drp1 to reduce T‐cell motility. To answer this point, we activated murine T cells *in vitro* for 48 h, and expanded them for additional 4d with IL‐2 (a timepoint in which we still observed PD‐1 surface expression, data not shown). Then, cells were preincubated for 2 h with BSA (as control) or PD‐L1 and then stimulated with CCL19 and CCL21 chemokines. Interestingly, while the chemokine stimulation promotes both Drp1 and ERK1/2 phosphorylation (Fig. 5A) and the mitochondrial fragmentation and relocation at the uropod (Fig. 5B), as expected [13], the concomitant engagement of the PD‐1 signaling prevents both Drp1 activation and mitochondrial relocation (Fig. 5B, C). Collectively, these data indicate that PD‐1 signaling engagement deprives T cells of a key cellular event (i.e., the Drp1‐dependent mitochondrial repositioning upon fragmentation) required to sustain motility.

Furthermore, we found that Drp1‐mediated motility is lost also in an *in vitro* generated exhausted‐like (T_ex_) state (obtained through induction of TCR hyporesponsiveness and without PD‐1 involvement, as described for Fig. S3C, D). Indeed, these cells, which migrate less than control functional T_eff_ cells (Fig. 5C), show an impaired Drp1 phosphorylation (Fig. 5D) and a lack of mitochondrial relocation at the uropod upon chemokine stimulation (Fig. 5E).

Last, we asked whether the rescue of Drp1 activity in tumor‐derived exhausted T cells is important for the effectiveness of anti‐PD‐1 immunotherapy in rescuing T‐cell motility. To answer this point, we isolated CD8^+^ TILs from MC38‐derived tumor‐bearing control and Drp1‐cKO mice treated with either anti‐IgG or anti‐PD‐1 antibodies. Interestingly, we found that anti‐PD‐1 treatment significantly increases motility of control PD‐1^pos^ CD8^+^ TILs (as assessed by Transwell assay), when compared to the motility of the same cells from anti‐IgG‐treated control mice (Fig. 5F). However, this effect is completely lost when looking at PD‐1^pos^ CD8^+^ TILs from Drp1‐cKO mice, whose T cells lack Drp1 (Fig. 5F). Of note, these data suggest that (a) the rescue of Drp1 activity by anti‐PD‐1 treatment is required to restore TIL motility and (b) Drp1 inhibition may be one of the mechanisms by which PD‐1 signaling reduces motility of PD‐1^pos^ CD8^+^ TILs.

Overall, these data indicate that PD‐1 signaling may mediate the reduction in CD8^+^ TILs motility via Drp1 downregulation, thus preventing the Drp1‐dependent mitochondrial fragmentation upon chemokine stimulation, a key step normally required for T‐cell motility.

### 3.6 PD‐1 does not modulate T‐cell metabolism through the regulation of Drp1 activity

Several works have shown that both the engagement of PD‐1 signaling and the downmodulation of Drp1 activity, during T‐cell activation, modulate both glycolysis and OXPHOS [13, 19, 29]. Given the here reported downregulation of Drp1 activation by PD‐1 signaling, we asked whether PD‐1 may exploit such a Drp1 downregulation to also modulate T‐cell metabolism. To answer this point, we analyzed the metabolism of both control and Drp1‐KO T cells upon activation in the presence of aCD3/28 or aCD3/28+PD‐L1 beads.

Interestingly, we found that upon 24 h of lymphocyte activation PD‐1 signaling, but not Drp1 ablation, reduces glycolysis, while the opposite is observed at 48 h (Fig. S5A, B). Therefore, although both PD‐1 engagement and Drp1 ablation can reduce glycolysis during T‐cell activation, their effects occur with different kinetics, Moreover, both PD‐1 engagement and Drp1 ablation alone partially reduce glucose uptake upon T‐cell activation, but the combination of the two further reduces glucose uptake (Fig. S5C). Regarding OXPHOS, while we observed no effects of PD‐1 engagement on basal respiration up to 48 h upon activation, this parameter is instead increased by Drp1 ablation at this timepoint (Fig. S5D, E), without a concomitant modulation of the mitochondrial membrane potential (Fig. S5F).

Overall, these data suggest that although both PD‐1 and Drp1 can modulate the T‐cell metabolism upon cell activation, and even if PD‐1 directly regulates the Drp1 activation status, the effects of PD‐1 signaling on metabolism likely do not depend on the concomitant modulation of Drp1 activity.

## 4 Discussion

It has been reported that tumor‐infiltrating T cell shows an altered mitochondrial functionality and morphology when compared with T cells from peripheral blood [30]. However, the modulation of mitochondrial morphology in different subpopulations of TILs has never been investigated before. Here, we report that in MC38‐derived tumors PD‐1^pos^ tumor‐infiltrating murine CD8^+^ T cells display a reduced activation of Drp1 and a more fused mitochondrial network when compared with PD‐1^neg^ counterparts. Of note, these data are shared also in a corresponding human context of colon tumor, in which tumor‐infiltrating lymphocytic elements almost never coexpress PD‐1 and active Drp1. Mechanistically, we provided evidence that PD‐1 signaling downregulates Drp1 activating phosphorylation on Ser616 (and consequently mitochondrial fragmentation) via the inhibition of ERK1/2 and mTOR kinases.

Also, we explored the functional consequences of such PD‐1‐dependent downregulation of Drp1 activity in the tumor context. Of the highest importance, altogether, our data suggest that PD‐1 signaling may exploit the downregulation of Drp1 activity to dampen some of the processes required for an optimal T‐cell functionality. In line with this, the restoration of Drp1 activity in TILs is strictly required for the effectiveness of the anti‐PD‐1 therapy. Specifically, Drp1 seems to play an important role in controlling both motility and proliferation of PD‐1^pos^ CD8^+^ TILs. Mechanistically, we observed that the Drp1‐dependent mitochondrial relocation at the cell rear‐edge during cell migration, a phenomenon occurring in healthy effector CD8^+^ T cells [13], is completely lost upon PD‐1 engagement in CD8^+^ T cells, which are unable to activate Drp1 upon chemokine stimulation. Our data are thus consistent with previous observations made in a persistent infection mouse model, in which the recovered motility of PD‐1^pos^ T cells upon anti‐PD‐1 treatment was associated with an increased activation of ERK [18], a kinase known to regulate Drp1 in T cells [12, 13]. Therefore, downregulation of ERK/Drp1 axis may be exploited by PD‐1 signaling to dampen T‐cell motility. Regarding the role of Drp1 in T‐cell proliferation, we previously reported that, in the absence of Drp1, T cells show an abnormal length of mitosis, due to the acquisition of aberrant centrosome morphologies [13], as observed also in cancer cells [31]. On the contrary, we here provided data suggesting that PD‐1 signaling does not regulate T‐cell metabolism via Drp1 downregulation. PD‐1 has been reported to dampen both glycolysis and OXPHOS upon cell activation in both murine and human T cells, although a detailed kinetic analysis of the metabolic modulations caused by PD‐1 engagement is still lacking. Although we here demonstrated that PD‐1 prevents Drp1 activation upon T‐cell stimulation, we found that in murine T cells early upon activation (i.e., up to 48 h) the effects on metabolism observed upon PD‐1 engagement and Drp1 ablation are either different in direction (PD‐1 does not modulate OXPHOS while Drp1 ablation increases it) or in timing (both PD‐1 and Drp1 ablation reduce glycolysis but at different timepoints).

Moreover, we could speculate that the downregulation of Drp1 by PD‐1 signaling may represent a mechanism exploited by exhausted TILs to modulate mitophagy, too. A defective mitophagy in PD‐1^positive^ T cells has been reported to contribute to the exhausted phenotype of these cells [32]. Interestingly, Drp1 may facilitate dismissal of small mitochondria via mitophagy [33]. Therefore, the downregulation of Drp1 activity in PD‐1^pos^ CD8^+^ TILs may provide a mechanism to reduce the mitophagy rate in these cells, preventing the generation of small mitochondria that can be targeted to degradation. This is consistent with the observation of a higher mitochondrial mass in CD8^+^ TILs [30].

Of note, a recent publication by Ogando *et al*. [29] shows an unaltered rate of mitochondrial fragmentation in stimulated T cells (independently of the presence of PD‐1 engagement) compared with unstimulated cells. This finding can be accounted for by significant differences in the methodology applied for the analysis. Indeed, they chose a specific parameter to estimate the mitochondrial fragmentation (i.e., circularity). At variance with that work, we considered here that stimulated T cells, compared to unstimulated (naïve) counterparts, are significantly larger, as a consequence of their activation *in vitro*, and also that the organelle circularity might consequently be altered, becoming a nonuseful parameter. Also, fused mitochondria in T cells appear more tangled due to the round shape of this nonadherent and small cell type. In addition, Ogando *et al*. only analyzed total levels of Drp1, without focusing on its specific phosphorylated residues [29], which are more reliable indicators of Drp1 activation compared with the total protein amount.

In sum, our data indicate that downregulation of Drp1 mediated by PD‐1 signaling may be required to attain an efficient inhibition of T‐cell response. Therefore, we dare to propose Drp1 as a therapeutic target to ameliorate exhausted T‐cell functionality during anticancer approaches, although drugs able to activate this protein need to be developed yet. Interestingly, CAR T‐cell‐based approaches are currently being exploited for the treatment of solid cancers [34], but they frequently fail to confer long‐term tumor regression due to a poor ability of CAR T cells to survive and infiltrate within a solid tumor mass. This may be partially explained by the tendency of CAR T cells to undergo functional exhaustion, similar to endogenous T cells [35]. However, whether a PD‐1‐dependent downregulation of Drp1 activity is also present in exhausted CAR T cells is still not known. Should such modulation be observed, targeting Drp1 activity in CAR T cells (either pharmacologically or genetically) could represent a new strategy to ameliorate CAR T‐cell survival or infiltration.

## 5 Conclusion

In sum, the modulation of Drp1 in tumor‐derived exhausted T cells may represent a valuable target to ameliorate anticancer immune response in a number of instances, and the manipulation of CAR T system to this aim may represent a valid future strategy.

## Conflict of interest

The authors declare no conflict of interest.

### Peer Review

The peer review history for this article is available at https://publons.com/publon/10.1002/1878‐0261.13103.

## Authors’ contribution

LS and SC conceived the work and wrote the manuscript, which has been approved by all authors. SC raised funding. LS and YA performed most of the experiments. GS helped to perform western blot experiments. VC and CT performed IHC on human tumor tissues. AC, CP, and GM performed and analyzed seahorse data. SM, BDA, and CQ provided blood and helped with metabolic experiments. All authors reviewed the results and approved the final version of the manuscript.

## Supporting information


**Fig. S1.** Further analysis on MC38‐derived murine tumors. Related to Figure 1.
**Fig. S2.** Functional analyses on activated T cells. Related to Figure 2.
**Fig. S3.** Further analyses of signaling pathways regulating the interaction between PD‐1 and Drp1. Related to Figure 3.
**Fig. S4.** Further analyses related to Drp1 conditional‐KO mice. Related to Figure 4.
**Fig. S5.** Analyses related to T cell metabolism.

## Data Availability

The data supporting the findings of this study are available from corresponding author upon reasonable request.
