# Supplementary material for: PD‐1‐induced T cell exhaustion is controlled by a Drp1‐dependent mechanism
Source: Mol Oncol. 2021 Oct 14;16(1):188–205. doi: 10.1002/1878-0261.13103 (PMC8732338; doi:10.1002/1878-0261.13103)
Supplement: Supplementary file 1 — Fig. S1. Further analysis on MC38‐derived murine tumors. Related to Figure 1. Fig. S2. Functional analyses on activated T cells. Related to Figure 2. Fig. S3. Further analyses of signaling pathways regulating the interaction between PD‐1 and Drp1. Related to Figure 3. Fig. S4. Further analyses related to Drp1 conditional‐KO mice. Related to Figure 4. Fig. S5. Analyses related to T cell metabolism. [file MOL2-16-188-s001.pdf]

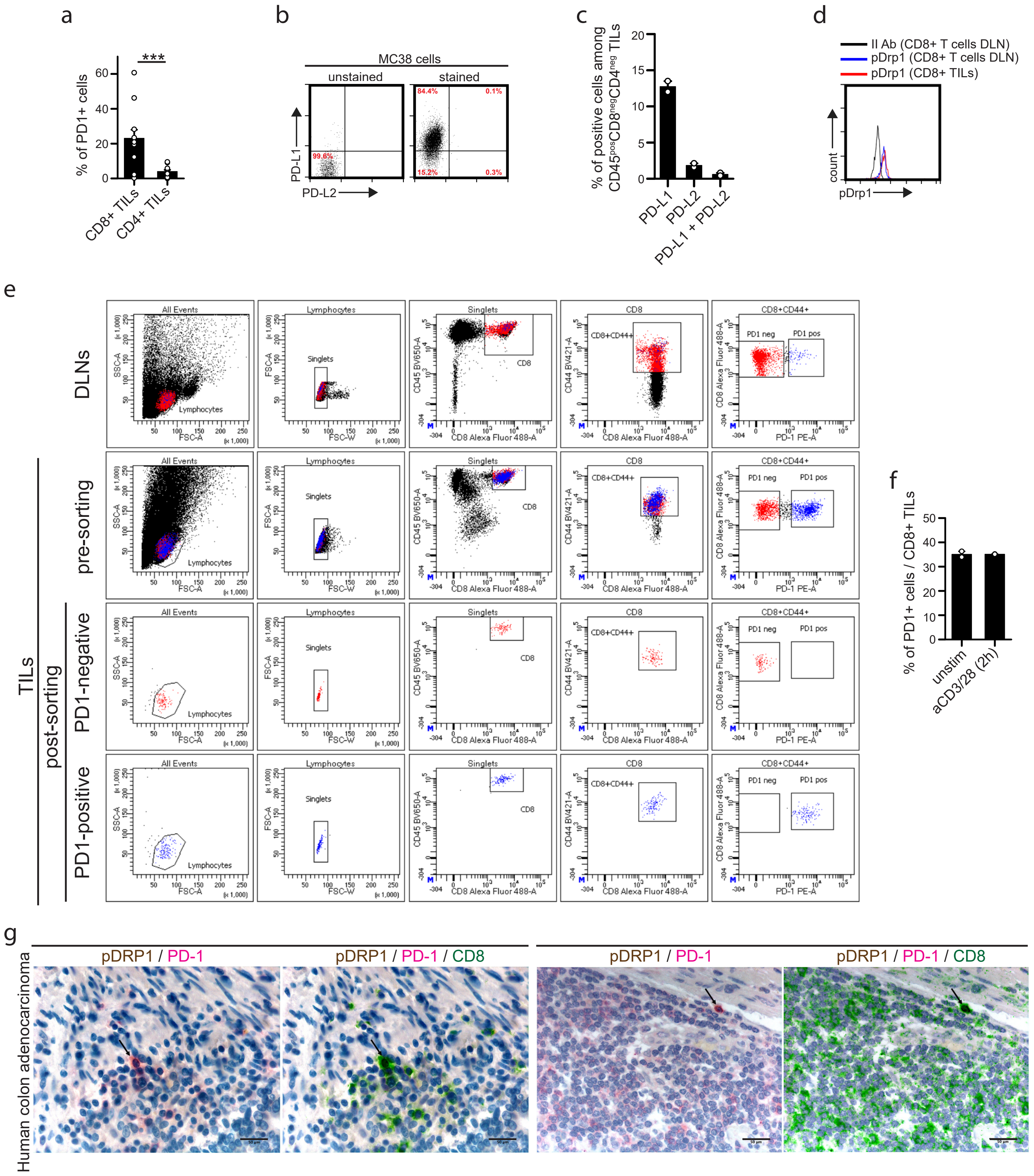

Supplementary Figure 1. Further analysis on MC38-derived murine tumors. Related to Figure 1.
(**a**) Percentage of PD-1+ cells among CD8+ and CD4+ T cells infiltrating MC38-derived tumors (n=12).
(**b**) Representive expression of PD-L1 and PD-L2 in MC38 tumor cells.
(**c**) Expression of PD-L1 and PD-L2 in CD45^{pos} non-T (i.e., CD4^{neg}CD8^{neg}) immune cells isolated from MC38-derived s.c. tumor masses grown for 18 days in WT c57BL/6 mice (n=2).
(**d**) Representative Drp1-pSer616 expression in CD8+ T cells from MC38-derived tumor mass (TILs) and draining lymph node (DLN) togheter with background signal of secondary antibody alone (IIAb) in DLN CD8+ T cells.
(**e**) Gating strategy to isolate CD45^{+}CD8^{+}CD44^{+} PD1^{neg} and PD1^{pos} T cells (used in Fig. 1b,c) from MC38-derived s.c. tumor masses grown for 18 days in WT c57BL/6 mice.
(**f**) Pecentage of PD1^{pos} cells among CD8^{+} TILs isolated from MC38-derived tumor masses (same as Figure 1b,c) and left unstimulated or stimulated 2h with aCD3/28-beads (n=2).
(**g**) Representative microphotographs of triple-marker immunohistochemistry for PD1 (rose), Drp1-pSer616 (pDrp1, brown) and CD8 (green) expression in lymphoid elements infiltrating human colon cancer.
Data are shown as mean ± SEM. Scale bar = 50μm. Significance is indicated as follows: *** = p<0.001.

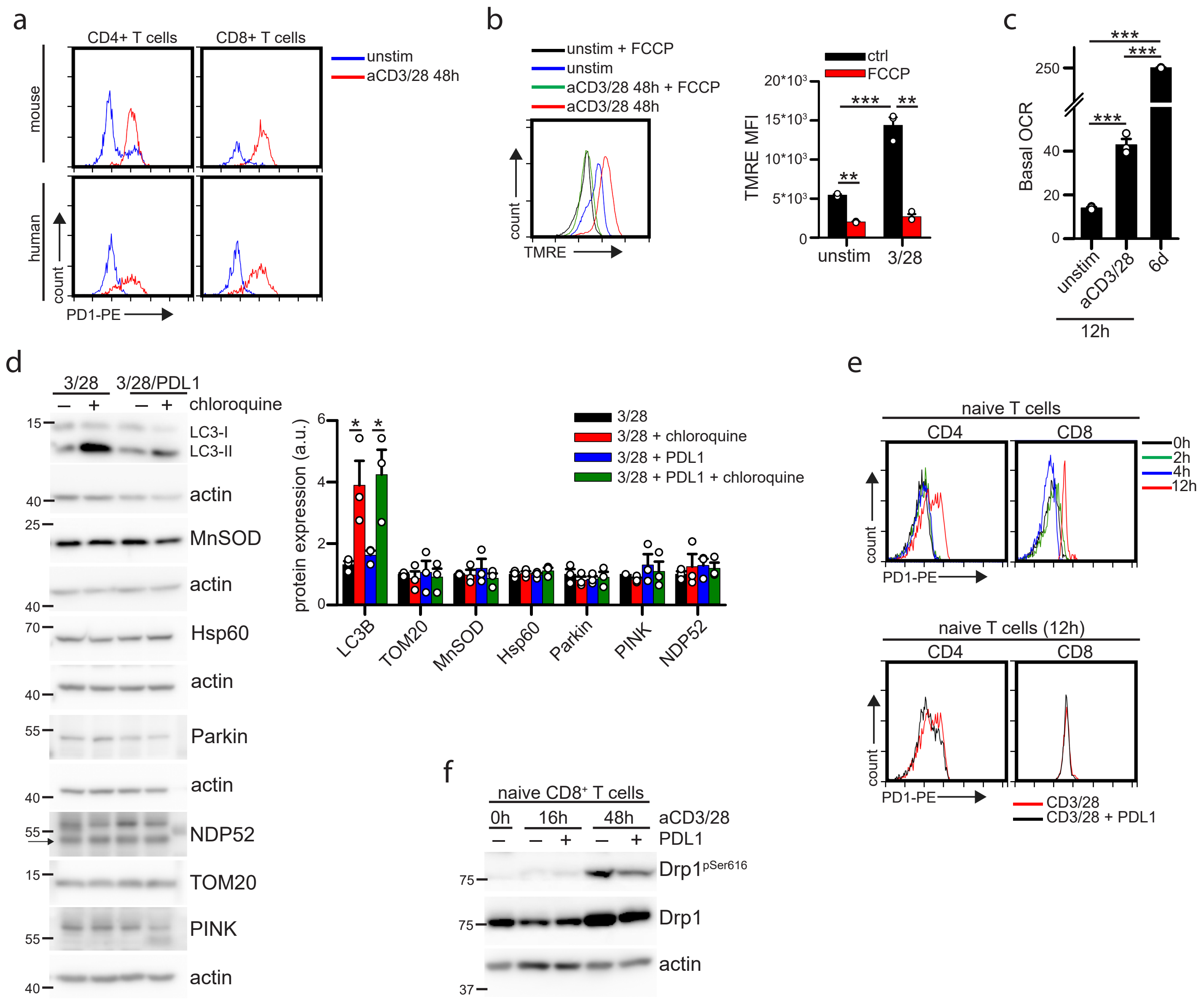

Supplementary Figure 2. Functional analyses on activated T cells. Related to Figure 2.

(**a**) Representative flow cytometry plots showing PD-1 expression in CD4+ and CD8+ T cells unstimulated (unstim) and stimulated for 48h with aCD3/28-coated beads. One experiment represenative of three different experiments.

(**b**) TMRE profile of murine WT T cells left unstimulated or stimulated for 48h with aCD3/28-coated beads. FCCP has been used as a positive control for depolarized mitochondria. Quantification of the TMRE mean fluorescence intensity (MFI) is reported in the graph on the right (n=3).

(**c**) Basal oxygen consumption rate (OCR) measured by seahorse in WT murine T cells unstimulated (12h unstim) or stimulated for 12h (12h 3/28) or stimulated for 48h and then expanded for 4 days in IL2-containing medium (6d) (n=3).

(**d**) Murine WT T cells have been isolated and stimulated in presence of anti-CD3/28- or anti-CD3/28+PDL1-beads for 48h. Chloroquine has been added for the last hour in culture. The relative expression levels of the indicated proteins are shown on the left and quantifications in the graph on the right (n=3).

(e) Evaluation of PD1 expression by flow cytometry in purified murine CD4^{+} and CD8^{+} naive T cells stimulated *in vitro* with beads coated with anti-CD3/CD28 (left) or anti-CD3/CD28 with or without PDL1 (right) for the indicated time.

(f) Representative western blot images showing Drp1 and Drp1^{pSer616} expression at the indicated time after stimulation in presence of anti-CD3/28- or anti-CD3/28-PDL1-beads in naive CD8+ T cells.

Data are shown as mean ± SEM. Significance is indicated as follows: * = p<0.05; ** = p<0.01; *** = p<0.001.

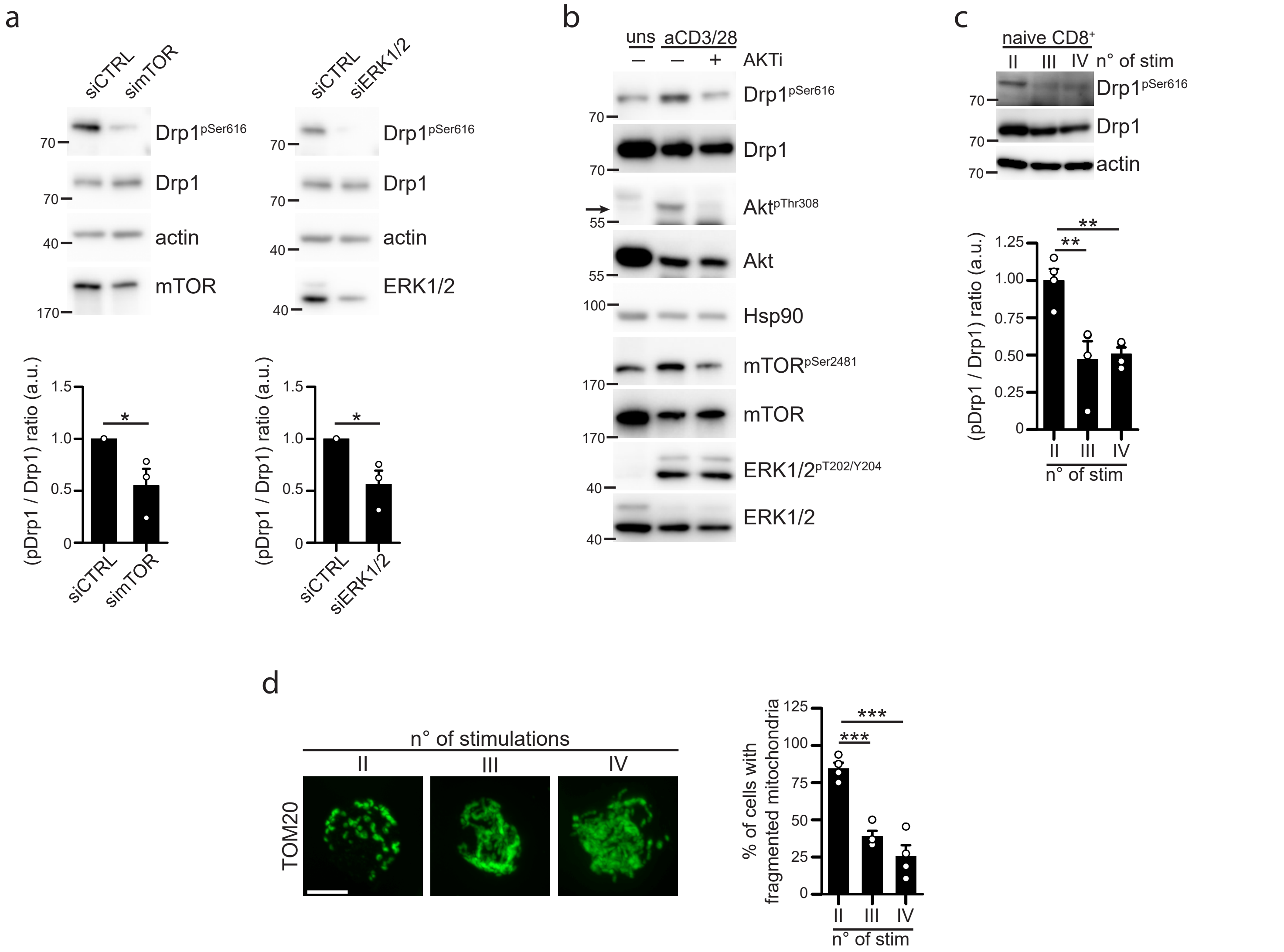

Supplementary Figure 3. Further analyses of signaling pathways regulating the interaction between PD-1 and Drp1. Related to Figure 3.

(**a**) Jurkat cells have been electroporated with siCTRL, siERK1/2 or simTOR and the expression levels of the indicated (phospho)-proteins has been evaluated by western blot (n=3).

(**b**) Murine T cells have been isolated from spleen of WT c57BL/6 mice and stimulated with anti-CD3/28-coated beads in presence or not of 1µM Akti-1/2 (AKT inhibitor: AKTi). Expression level of the indicated (phospho)-proteins has been assessed by western blot after 4h (pmTOR, pAkt, pERK) and 48h (pDrp1) (data show one experiment representative of three independent experiments).

(**c-d**) Murine CD44^{neg} naïve CD8+ T cells have been isolated from spleen of WT c57BL/6 mice and stimulated up to 4 times with plate-coated anti-CD3 Ab plus soluble anti-CD28 for 24h. After each stimulation, cells were left to recover 6 days in IL2-containing medium before the next stimulation. Immediately after the second (II), third (III) and fourth (IV) stimulationIn (c), the expression level of the indicated (phospho)-proteins has been evaluated by western blot (c, n=4) and the mitochondrial network analysed through anti-TOM20 staining (d). Quantification of the percentage of cells showing fragmented mitochondria in each condition is reported in the graph on the right (d, n=4).

Data are shown as mean ± SEM. Scale bar: 5µm in **d**. Significance is indicated as follows: *=p<0.05; **=p<0.01; ***=p<0.001.

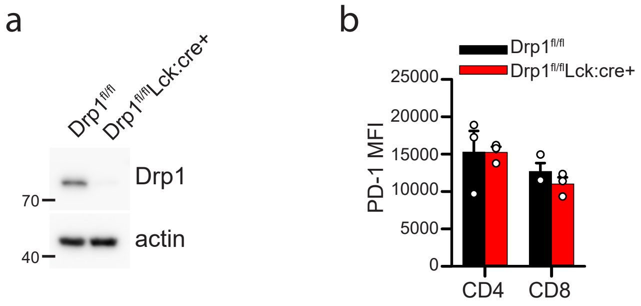

Supplementary Figure 4. Further analyses related to Drp1 conditional-KO mice. Related to Figure 4.
(**a**) Representative western blot for Drp1 expression in T cells isolated from spleen of $Drp1^{fl/fl}$ and $Drp1^{fl/fl}$Lck:cre+ mice.
(**b**) PD-1 expression (median fluorescent intensity, MFI) in control ($Drp1^{fl/fl}$) and Drp1-KO ($Drp1^{fl/fl}$Lck:cre+) T cells stimulated for 48h *in vitro* with aCD3/28-beads (n=3).
Data are shown as mean ± SEM.

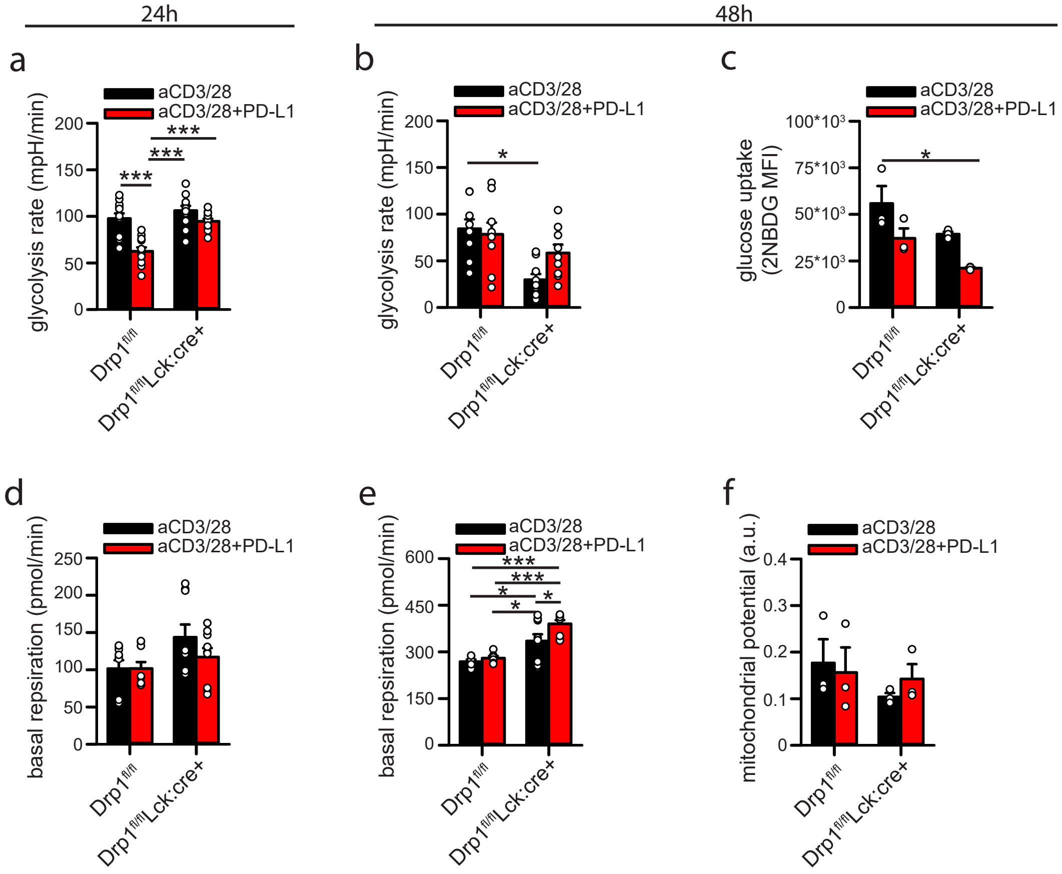

Supplementary Figure 5. Analyses related to T cell metabolism.
(**a**-**f**) Control (Drp1$^{fl/fl}$) and Drp1-KO (Drp1$^{fl/fl}$Lck:cre+) T cells have been isolated from mouse spleen and stimulated *in vitro* for the indicated time (24h or 48h) with aCD3/28- or aCD3/28+PD-L1-beads. The following parameters have been measured by seahorse: basal glycolysis at 24h (a, n=12) and 48h (b, n=12); basal respiration at 24h (d, n=9) and 48h (e, n=6). Furthermore, glucose uptake has been assessed at 48h as 2-NBDG MFI (c, n=3) and mitochondrial membrane potential as (TMRE / MitoTrackerGreen) ratio (f, n=3).
Data are shown as mean ± SEM. Significance is indicated as follows: *=p<0.05; ***=p<0.001.
